# REVERBa couples the circadian clock to hepatic glucocorticoid action

**DOI:** 10.1172/JCI96138

**Published:** 2018-09-04

**Authors:** Giorgio Caratti, Mudassar Iqbal, Louise Hunter, Donghwan Kim, Ping Wang, Ryan M. Vonslow, Nicola Begley, Abigail J. Tetley, Joanna L. Woodburn, Marie Pariollaud, Robert Maidstone, Ian J. Donaldson, Zhenguang Zhang, Louise M. Ince, Gareth Kitchen, Matthew Baxter, Toryn M. Poolman, Dion A. Daniels, David R. Stirling, Chad Brocker, Frank Gonzalez, Andrew S.I. Loudon, David A. Bechtold, Magnus Rattray, Laura C. Matthews, David W. Ray

**Affiliations:** 1 Faculty of Biology, Medicine and Health, University of Manchester, Manchester Academic Health Sciences Centre, and Specialist Medicine, Central Manchester Foundation Trust, Manchester, United Kingdom.; 2 Laboratory of Metabolism, Center for Cancer Research, National Cancer Institute, NIH, Bethesda, Maryland, USA.; 3 Biopharmaceutical Molecular Discovery, GlaxoSmithKline, Medicines Research Centre, Stevenage, United Kingdom.; 4 Leeds Institute of Cancer and Pathology, Faculty of Medicine and Health, University of Leeds, Leeds, United Kingdom.; 5 Oxford Centre for Diabetes, Endocrinology and Metabolism, University of Oxford, Oxford, United Kingdom.

**Keywords:** Endocrinology, Metabolism, Transcription

## Abstract

The glucocorticoid receptor (GR) is a major drug target in inflammatory disease. However, chronic glucocorticoid (GC) treatment leads to disordered energy metabolism, including increased weight gain, adiposity, and hepatosteatosis — all programs modulated by the circadian clock. We demonstrated that while antiinflammatory GC actions were maintained irrespective of dosing time, the liver was significantly more GC sensitive during the day. Temporal segregation of GC action was underpinned by a physical interaction of GR with the circadian transcription factor REVERBa and co-binding with liver-specific hepatocyte nuclear transcription factors (HNFs) on chromatin. REVERBa promoted efficient GR recruitment to chromatin during the day, acting in part by maintaining histone acetylation, with REVERBa-dependent GC responses providing segregation of carbohydrate and lipid metabolism. Importantly, deletion of *Reverba* inverted circadian liver GC sensitivity and protected mice from hepatosteatosis induced by chronic GC administration. Our results reveal a mechanism by which the circadian clock acts through REVERBa in liver on elements bound by HNF4A/HNF6 to direct GR action on energy metabolism.

## Introduction

Glucocorticoids (GCs; cortisol in humans, corticosterone in rodents) are critical regulators of energy metabolism and immunity. Their secretion by the adrenal gland follows a circadian pattern, with serum concentrations peaking before the active phase (day in humans, night in rodents). Synthetic GCs are the most potent antiinflammatory agents known and are widely used therapeutically, with more than 1% of the population in the United Kingdom holding a prescription long-term. However, frequent therapeutic use is accompanied by development of severe side effects, notably fat accumulation, hyperglycemia, and hepatosteatosis (1). Inactive GC receptor (GR) is bound by ligand in the cytoplasm and undergoes translocation to the nucleus, where it binds GC response elements (GREs) in the genome to either enhance or repress gene transcription. Mechanisms to explain how the same molecule can drive gene activation or repression remain under investigation, but likely require an allosteric change induced by DNA target sequence and/or co-binding with other transcription factors. For gene activation, homodimeric GR recruits coactivator molecules including steroid receptor coactivators (SRC1–3) and histone acetyltransferases (CBP/p300) (2, 3). In contrast, gene repression involves tethering of GR to transcription factors such as the inflammatory regulator NF-κB and in doing so interferes with that factor’s transactivation mechanism (4–6), or direct binding to negative GREs to recruit repressing cofactors (7, 8).

The subject of cell type–specific GR action is attracting increasing attention, and the existence of “primed” enhancer sites directed by tissue-specific transcription factor activity has emerged as an important determinant of GC effects (9, 10). This work has identified transcription factors that can serve as pioneer factors for GR, such as AP-1, which drives a state-dependent change in the GR cistrome in activated macrophages (11), and the hepatocyte-specific transcription factors HNF4A and HNF6, which do the same in liver (12). In this way GR and other nuclear receptors have preprogrammed cistromic profiles in target cells that await appropriate environmental cues, such as hormone signals.

Circadian rhythms in mammals are built and governed by a complex hierarchy of molecular and cellular interactions. Historically, the central hypothalamic suprachiasmatic nucleus (SCN) pacemaker has been viewed as pivotal to systemic synchrony, but recent studies now clearly demonstrate that peripheral organs can maintain tight local synchrony divergent from the SCN in response to feeding and metabolic cues, and a range of environmental signals (13–15). At the molecular level, the circadian clock is conserved in all cell types and is driven by a rhythmic feed-forward transcriptional/translational loop (BMAL1, CLOCK/NPAS2) and rhythmic negative feedback (Cry1/2, Per1/2, and the nuclear receptors REVERBa and REVERBb). REVERBa and REVERBb constitute a negative feedback loop to stabilize core clock oscillation. In the liver, REVERBs have a major role in conferring clock control to lipogenic gene expression (15), and recent discoveries identify both DNA binding regulation of the core clock and DNA binding–independent regulation of energy metabolism as distinct mechanisms of action. The latter requires HNF6/HNF4A factors to tether REVERBa to the genome (16, 17). We now know that the circadian clock is a potent regulator of both metabolism and immunity (18–21). Disruption of clock genes (*Bmal1*, *Clock*, *Cry*, *Reverb*) has been shown to profoundly alter energy metabolism and dysregulate inflammatory responses — all of which are also affected by GR. Close coupling between cellular clock machinery and GR is supported by protein interactions between CRY and GR to affect carbohydrate metabolism, and by the potent clock-entraining activity of the GR, which transactivates *Per2* (22). An intriguing recent report also suggested functional crosstalk between the GR and REVERBa, although the mechanism and physiological consequences of such an interaction were not delineated (23). Furthermore, studies in the rat suggest that timing of exogenous GC dosing impacts liver lipid metabolism, but again the underlying mechanism was not determined (24). These timing-dependent phenomena are interesting, especially because the CRY:GR crosstalk mechanism primarily affected carbohydrate metabolism, suggesting the existence of multiple points of circadian clock:GR coregulation. Despite this, there has been limited application of timing information in GC therapy, with nighttime release of prednisolone offering additional therapeutic effect (25). As so little is known about how biological time affects GR function, we investigated circadian control of GC action.

We reveal a major time-of-day gating in the spectrum of GC-regulated genes when mice are treated in the day or night. This temporal dynamic is especially pronounced in metabolic (liver) versus nonmetabolic (lung) tissues, and segregates carbohydrate and lipid metabolic processes. Deletion of *Reverba* caused a profound shift in both the makeup and time dependency of GC-regulated gene expression. Focusing on the liver, we identify a high prevalence of closely related genomic binding sites for GR and REVERB transcription factors, as well as direct physical interaction of GR with REVERBa. Importantly, chronic GC dosing in mice drove a shift in liver lipid metabolism, with only a minor impact on carbohydrate metabolism. The sparing of *Reverba*-KO (REVERBaKO) mice from GC-induced hepatosteatosis marks the physiological importance of the GR-REVERB interaction. These findings are significant for the development of chronotherapy using GC, as for example the recently launched Lodotra modified release prednisolone preparation (25), which delivers active GC to patients at night (equivalent to the mouse day).

## Results

### Time of day determines GC action.

**Figure 1:**
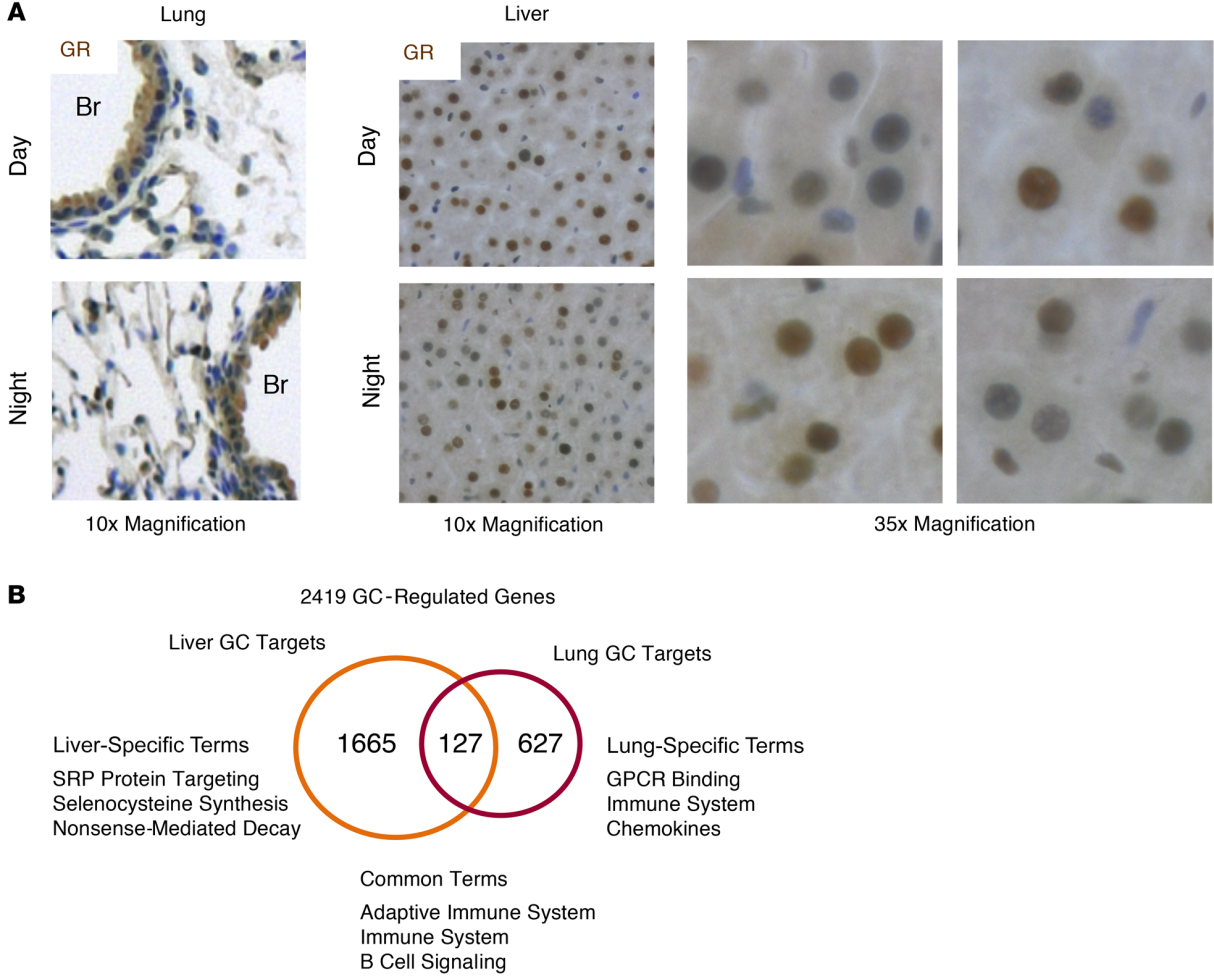
GCs induce tissue-specific transcriptomes. (**A**) GR immunohistochemistry in lung and liver at ZT8 (Day) and ZT20 (Night). GR expression is shown in brown; nuclei are blue. Br, bronchioles. C57BL/6 mice were given vehicle or 1 mg/kg i.p. dex at ZT6 (1 pm, day) or ZT18 (1 am, night) and culled 2 hours later, and lung and liver were analyzed by RNA-Seq. (**B**) Venn diagram depicting all GC-regulated genes identified byDESeq2 (*n* = 2 per group, >2-fold change to vehicle control, <0.05 FDR). Lung- and liver-specific targets are indicated, with gene ontology terms for each group listed below. SRP, signal recognition particle.

We first defined the time dependency of acute GC responses using the synthetic GC dexamethasone (dex; 1 mg/kg) in nonmetabolic (lung) and metabolic (liver) tissue. Secretion of endogenous GC follows a circadian pattern, with peak serum concentrations occurring before the active phase (night in rodents). Dex administration was therefore timed to the middle of the day (zeitgeber time 6 [ZT6], 1:00 pm) or the middle of the night (ZT18, 1:00 am), when endogenous corticosterone concentrations are similar (Supplemental Figure 1A; supplemental material available online with this article; https://doi.org/10.1172/JCI96138DS1). We also analyzed expression and localization of GR in the lung and liver, and we did not observe substantial changes in GR expression or nuclear localization between these time points (Figure 1A; and Supplemental Figure 1, B and C, P** = NS). Recent data have shown a change in GR phosphorylation and translocation to the nucleus by time of day, but critically, this change accompanies the diurnal change in serum corticosterone and likely reflects the activation of GR by binding to its endogenous ligand. It is noteworthy that our studies were performed at the time points when serum corticosterone, GR phosphorylation at S275 and S212, and nuclear GR abundance are similar in mouse liver (26, 27). We harvested total RNA from lung and liver of vehicle- and dex-treated mice at ZT6 and ZT18 (Figure 1B). RNA was analyzed by RNA sequencing (RNA-Seq), and across tissues and time points 2,419 GC-regulated genes were identified. Of these, the majority were tissue specific (627 genes in lung, 1,665 genes in liver). There was a restricted set of common targets (127 genes), and these were associated with antiinflammatory GC effects.

**Figure 2:**
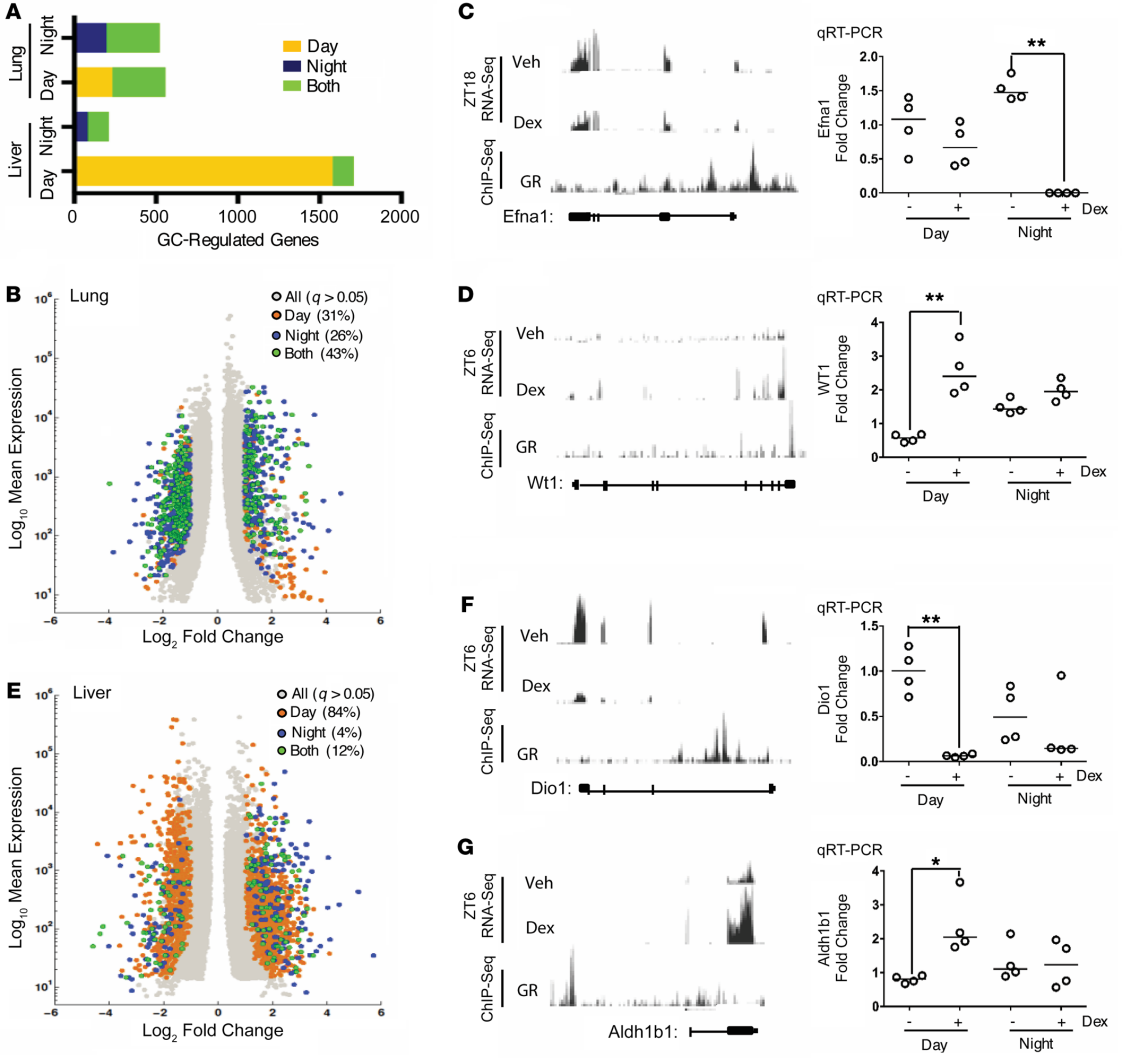
GC sensitivity in liver is regulated by time of day. (**A**) Histogram depicting number and time-of-day regulation of GC targets for each tissue. (**B**) Base mean expression versus log_2_ fold change plots for GC-regulated genes in lung show direction of regulation. (**C** and **D**) Two time-specific exemplars were validated by qRT-PCR (Veh and dex), and GR DNA binding (GR) was assessed via analysis of GR ChIP-Seq data. (**E**) Base mean expression versus log_2_ fold change plots for GC-regulated genes in liver show direction of regulation. (**F** and **G**) Two time-specific exemplars were validated by qRT-PCR, and GR DNA binding was assessed using GR ChIP-Seq data. For qRT-PCR data, individual data points are shown with median (*n* = 4 per group). Statistical analysis by Kruskal-Wallis test with a Dunn’s multiple comparisons correction, where *P < 0.05, **P < 0.01. Volcano plots depict all GC-regulated genes identified byDESeq2 (*n* = 2 per group, >2-fold change to vehicle control, <0.05 FDR).

In lung, a similar total number of genes were responsive to GC at the 2 administration times, but of these, only 43% were responsive to dex at both time points, indicating a temporal remodeling of the GC response (Figure 2A). Gene ontology analyses revealed antiinflammatory pathways as highly GC regulated at both times (Supplemental Tables 1 and 2), with transactivated and transrepressed genes equally represented (Figure 2B). RNA-Seq tracks were compared with GR ChIP sequencing (ChIP-Seq) tracks from ZT4 to confirm direct GR targets, and time-of-day effects were confirmed by quantitative real-time PCR (qRT-PCR) for Efna1, a night-specific GC target, and Wt1, a day-specific GC target (Figure 2, C and D).

Timing of administration had a major effect on GC sensitivity in the liver, with 1,709 genes responsive to daytime GC administration and only 211 genes regulated at night (Figure 2E). As in lung, similar proportions of transactivated and transrepressed genes were observed at both times. Again, the RNA-Seq tracks were compared with GR ChIP-Seq tracks at ZT4, and the time-dependent switch in GC sensitivity was validated by qRT-PCR for Dio1 and Aldh1b1 (Figure 2, F and G).

Our data analysis revealed an excess of daytime GC-regulated genes in the liver. KEGG pathway analyses revealed a strong time of day–dependent regulation of energy metabolism, including carbohydrate, lipid, pyruvate, and oxidative phosphorylation (Supplemental Figures 2 and 3, Supplemental Tables 3 and 4). These analyses highlighted a GC-dependent impact on mitochondrial function in a time of day–specific manner. We therefore analyzed mitochondrial mass and bioenergetic metabolite concentrations in livers from GC-treated mice by day and by night. A profound GC-induced loss of mitochondrial mass was observed in liver, but only in animals treated in daytime (Supplemental Figure 3B), when AMP levels rise in response to the fasted state. A change in mitochondrial mass did not occur in lung (Supplemental Figure 3C). The AMP concentration showed a small but significant increment in response to GC in the day, but no response at night, when the mice are in an energetically replete state and AMP concentrations are low (Supplemental Figure 3, D and E). There was also a sharp fall in the NAD^+^/NADH ratio in response to GC treatment in the day and no change observed at night (Supplemental Figure 3F). No differences in ATP or ADP concentrations were seen by time of day or treatment status (Supplemental Figure 3D), indicating that the altered mitochondrial mass is compensated by greater ATP synthesis efficiency.

### Identification of REVERB, and CRY co-binding with GR in the hepatic genome.

**Figure 3:**
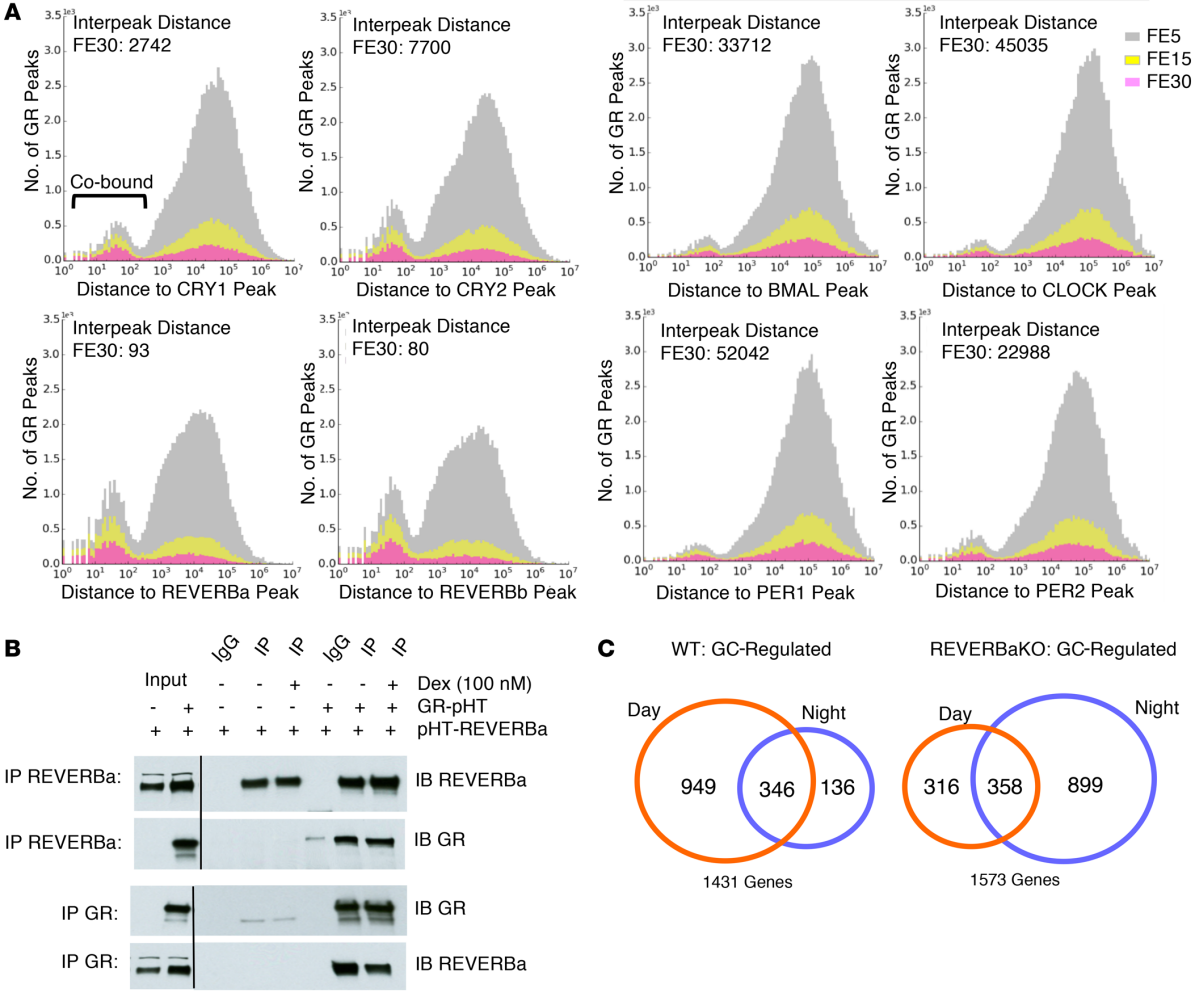
REVERBa regulates GR function. (**A**) Co-binding histograms depict the distance between GR binding events and the nearest clock transcription factor ChIP-Seq summit, using 3 stringencies (FE scores). Median interpeak distances for the highest stringency (FE30) is shown. (**B**) Coimmunoprecipitation of epitope-tagged REVERBa and GR. C57BL/6 (WT) and REVERBaKO mice were given 1 mg/kg i.p. dex at ZT6 (day) or ZT18 (night) and culled 2 hours later, and livers were analyzed by RNA-Seq. (**C**) Venn diagram depicts all GC-regulated genes identified byDESeq2 (*n* = 5 per group, >2-fold change to vehicle control, <0.1 FDR). pHT, polyhistidine tag.

The time-dependent switch in GC sensitivity in liver suggests the operation of a circadian control mechanism capable of targeting the GR. The chromatin loading of these core clock transcription factors shows strong daily variation (based on data reported in ref. 28, Supplemental Figure 4, A and B), and there is evidence that the CRY proteins bind GR to direct GR control of carbohydrate metabolism in the liver (29). To investigate the role of CRY in conferring the time-of-day variation in GC response, and to identify additional circadian crosstalk with GR, we determined the proximity of the mapped cistrome of GR, obtained at ZT4, to each of the circadian transcription factors in liver (Figure 3A). Putative co-binding was defined as a distance of less than 120 bp between ChIP-Seq summits and high-stringency (fold enrichment 30 [FE30] for GR data) co-bound sites, indicating a degree of cooperative binding (co-binding delineated in the leftmost peak in Figure 3A). Pooled data from across the circadian cycle were used in order to avoid potential complications arising from the different phases of core circadian transcription factor expression in mouse liver (28). The level of co-binding was quantified by determining the ratio of co-binding events (interpeak distance <120 bp) to non-proximal binding events (interpeak distance >120 bp). As it compares the relative abundance of proximal and non-proximal binding, the approach is not sensitive to differences in the total number of transcription factor binding peaks seen between analyzed cistromes (Supplemental Figure 4, C–F).

Co-binding of GR and CRY was revealed, consistent with the previously reported physical interaction between GR and CRY (29), and this observation provided useful confirmation that our analytical approach was capable of detecting a previously determined co-binding event. In addition, we discovered a surprisingly high frequency of co-binding of GR with REVERBa and REVERBb, with median interpeak distances of only 93 and 80 bp, respectively (Figure 3A). Notably, the REVERBa and REVERBb cistromes were generated by different antibodies and showed a high degree of overlap (Supplemental Figure 4E), as would be expected, as the DNA binding specificities of the two transcription factor paralogs show near identity (16). Other core clock transcription factors (BMAL1, CLOCK, PER1/2), however, showed little co-binding with GR.

To investigate the contribution of time-of-day variation in circadian transcription factor expression and function to our co-binding analysis, we separated the CRY1 and CRY2 cistromes by time, and analyzed GR-CRY co-binding with GR cistromes generated at 6:00 am and 6:00 pm. (Supplemental Figure 4, F and H). The co-binding of GR and CRY was seen at all the time points analyzed and followed the pattern of DNA loading of the CRYs. Similarly, analysis of REVERBa and REVERBb cistromes with GR binding at 6:00 a.m. or 6:00 p.m. confirmed our findings of a high incidence of co-binding with GR (Supplemental Figure 4G).

The observation, made using computational approaches, that REVERBs and GR bind in close proximity to selected sites in the genome suggests that the two transcription factors interact. To investigate this possibility, we set out to use a different approach to test REVERBa interaction with GR, via coimmunoprecipitation. Using expression of tagged GR and REVERBa in HEK cells, we observed a strong and specific interaction between the two nuclear receptors (Figure 3B and Supplemental Figure 10). Interestingly, we did not see a ligand dependence in the interaction between the two nuclear receptors, implying that the GR interaction surface is not the ligand-binding domain. We went on to investigate coimmunoprecipitation of endogenous proteins in the liver. We selected the time point of maximal endogenous REVERBa protein expression but were unable to confidently identify an interaction. This may reflect the low expression level of the two endogenous nuclear receptors and the dynamic interactions that occur on chromatin (Supplemental Figure 11).

### REVERBa expression is required to maintain appropriate time-of-day GR action in the liver.

The discovery that GR and REVERBa can exist within the same molecular complex implies an important functional crosstalk between the two receptors. To investigate this possibility, we examined GC responses in mice lacking *Reverba* and littermate controls. The loss of REVERBa caused a dramatic change in the temporal characteristics of GC response, with loss of many daytime-responsive genes, and acquisition of additional GC targets at night (Figure 3C).

**Figure 4:**
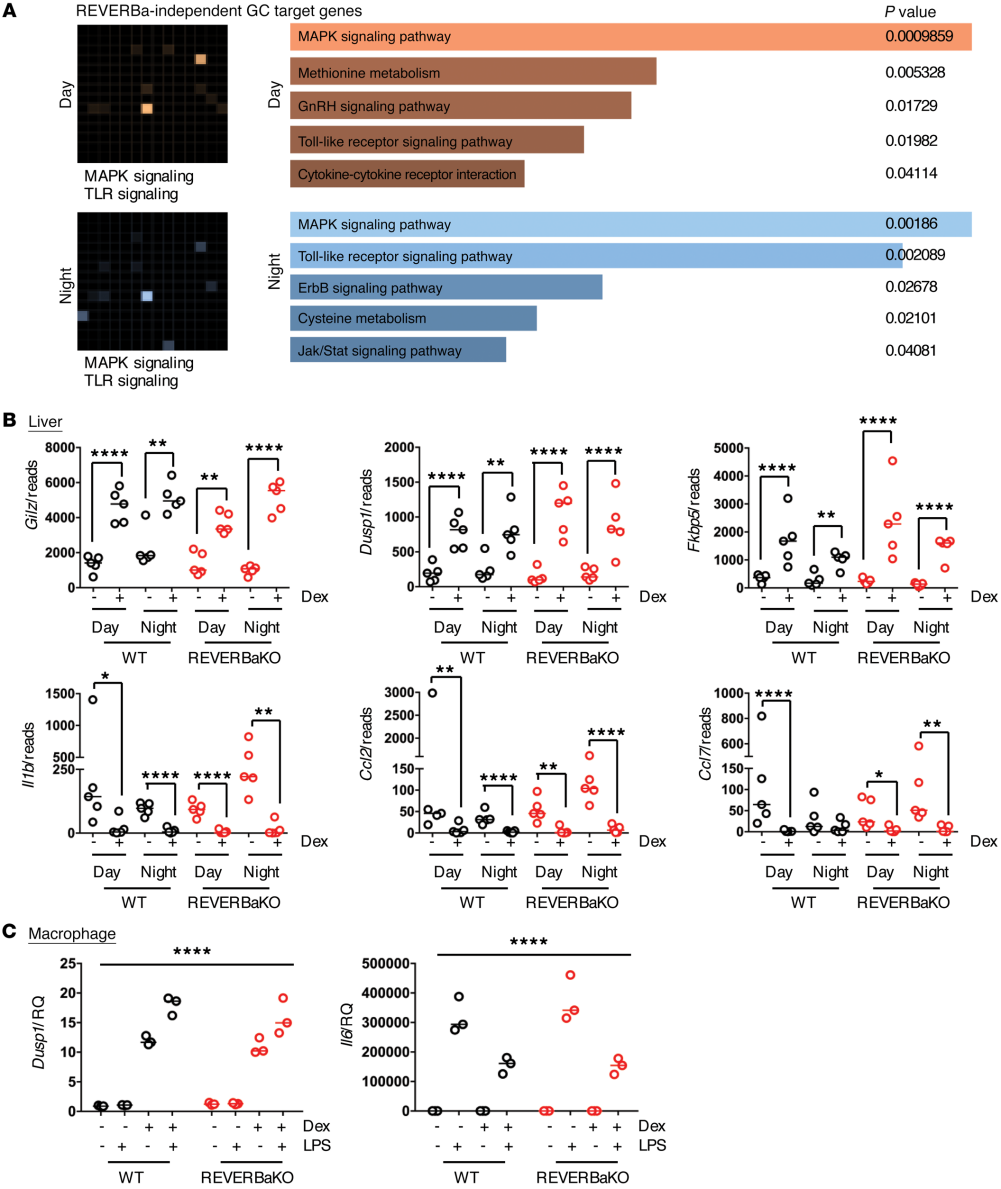
REVERBa does not regulate antiinflammatory GC effects. (**A**) Gene ontology (Enrichr) grids for REVERBa-independent GC targets with the 2 highest-ranking terms listed underneath. (**B**) Graphs show RNA-Seq reads for antiinflammatory GC target genes from liver RNA-Seq. Individual samples (*n* = 5) are plotted with the median for each group. Bone marrow–derived macrophages were isolated from REVERBaKO and WT littermate control mice, treated with vehicle or 100 nM dex for 1 hour, then with 100 ng/ml LPS for 4 hours. (**C**) GC regulation was determined by qRT-PCR for *Dusp1* and *Il6*; no genotypic differences were observed; data shown as median. Two-way ANOVA (macrophages, *n* = 3) effect of treatment, *P* < 0.001; Fisher’s exact test adapted for negative binomial distribution (RNA-Seq), *q < 0.05, **q < 0.01, ****q < 0.0001. RQ, relative quantity.

Gene ontology analysis using Enrichr highlighted that genes consistently responsive to GC signaling (irrespective of time of day or of REVERBa deletion) were highly enriched for the antiinflammatory actions of GR (Figure 4A). We therefore examined the actions of GR on index inflammatory target genes in the liver and isolated immune populations of REVERBaKO and WT mice (Figure 4B). We demonstrate that induction of antiinflammatory effectors *Gilz* and *Dusp1*, and repression of the proinflammatory effectors *Il1b* and *Ccl2* by GC is not under REVERBa control. We extended our analysis to isolated bone marrow–derived macrophages activated in vitro with LPS (Figure 4C) and further show that loss REVERBa had no effect on the potent antiinflammatory actions of GR stimulation.

**Figure 5:**
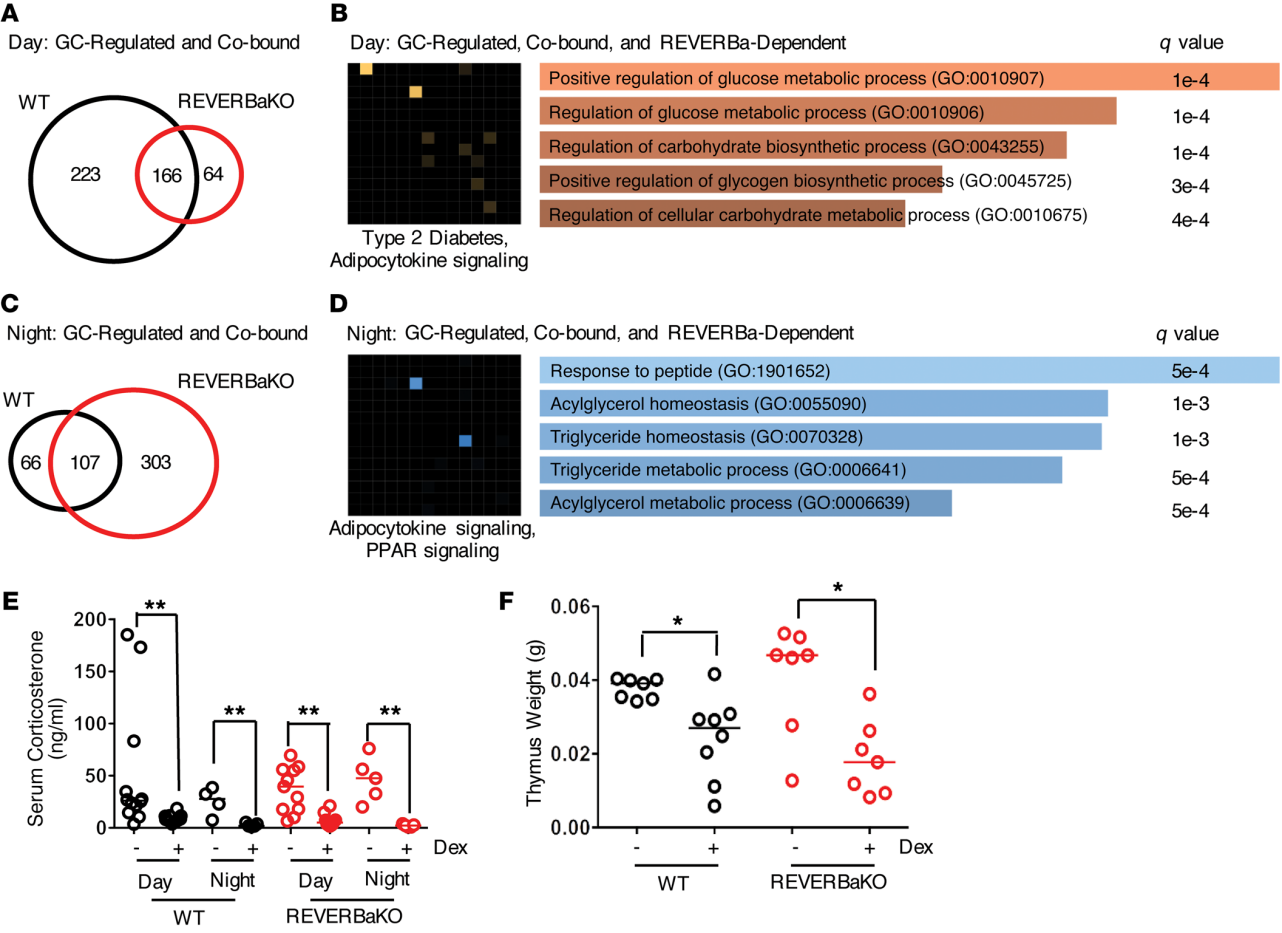
REVERBa selectively regulates GC action depending on time of day. (**A**) Daytime-regulated GC targets were further stratified for regions of GR-REVERBa co-binding from Figure 3. (**B**) Gene ontology (Enrichr) of GC-regulated, co-bound, REVERBa-dependent genes in the day. (**C**) Nighttime-regulated GC targets were further stratified for regions of GR-REVERBa co-binding from Figure 3. (**D**) Gene ontology (Enrichr) of GC-regulated, co-bound, REVERBa-dependent genes at night. (**E**) Dex suppression test via measurement of serum corticosterone at both ZT6 and ZT18 in WT and REVERBaKO mice with a single dose of dex 1 mg/kg. C57BL/6 (WT) and REVERBaKO mice were given 1 mg/kg i.p. dex or vehicle at ZT6 every 48 hours for 8 weeks. (**F**) C57BL/6 (WT) and REVERBaKO mice were given 1 mg/kg i.p. dex or vehicle at ZT6 every 48 hours for 8 weeks. Thymus weight was measured. Corticosterone measurement, *n* = 5–12; thymus weight, *n* = 7–8, *P < 0.05, **P < 0.01, Mann-Whitney *U* test; shown as median.

Analysis of gene regulation by GC in WT mice showed a dramatic loss in the responsiveness of genes involved in carbohydrate and lipid metabolism at night (Supplemental Figure 2). Again, REVERBaKO animals were used to assess the impact of REVERBa on the time-of-day segregation of GC action. We first identified daytime GC-regulated target genes in both genotypes and further enriched this gene set by identifying genes close to GR/REVERBa co-bound peaks (derived from analysis presented in Figure 3A). In WT mice, 223 genes were identified close to GR/REVERBa co-bound sites, which were exclusively GC responsive in the day. A set of 64 such genes were identified only in REVERBaKO animals (Figure 5A). Gene ontology analysis of the combined set of genes that either lose or gain GC response by day identified carbohydrate metabolic processes as being highly enriched (Figure 5B). Therefore, we propose that the loss of GC gene regulation observed in the liver of REVERBaKO mice during the day will result in altered carbohydrate metabolic responses to GC, by affecting the pathways identified and mapped in Supplemental Figure 2.

We next examined nighttime-specific GC-dependent gene regulation. GC treatment of WT mice at night only regulated 66 genes with putative co-binding targets; however, the REVERBaKO mice acquired 303 new GC gene targets (Figure 5C). Gene ontology analysis of this combined set of lost and gained genes by genotype revealed strong enrichment of lipid metabolic genes (Figure 5D). Therefore, the gain of nighttime GC-regulated genes in the REVERBaKO mice is predicted to result in an impact on GC regulation of hepatic lipid metabolism.

### REVERBa preferentially couples GR to the hepatic lipid metabolism program.

In previous work, functional interactions between CRY and GR were identified (29). In CRY1/2 double-KO mice there was a striking loss of GC suppression of the hypothalamic-pituitary-adrenal axis, with persistent high circulating corticosterone levels observed in some animals after an injection with the potent synthetic GC dex. We did not see any differences in corticosterone suppression by time of day or *Reverba* genotype (Figure 5E).

Long-term GC treatment drives abnormal carbohydrate and lipid metabolism, and we therefore investigated the impact of GR/REVERBa crosstalk on the metabolic consequences of chronic GC treatment. WT and REVERBaKO littermates were treated with dex, 1 mg/kg by i.p. injection, at ZT6 every 48 hours for 8 weeks (29). There was marked thymic atrophy to a similar degree in both genotypes (Figure 5F), further supporting a lack of crosstalk between GR and REVERBa in immune regulation.

**Figure 6:**
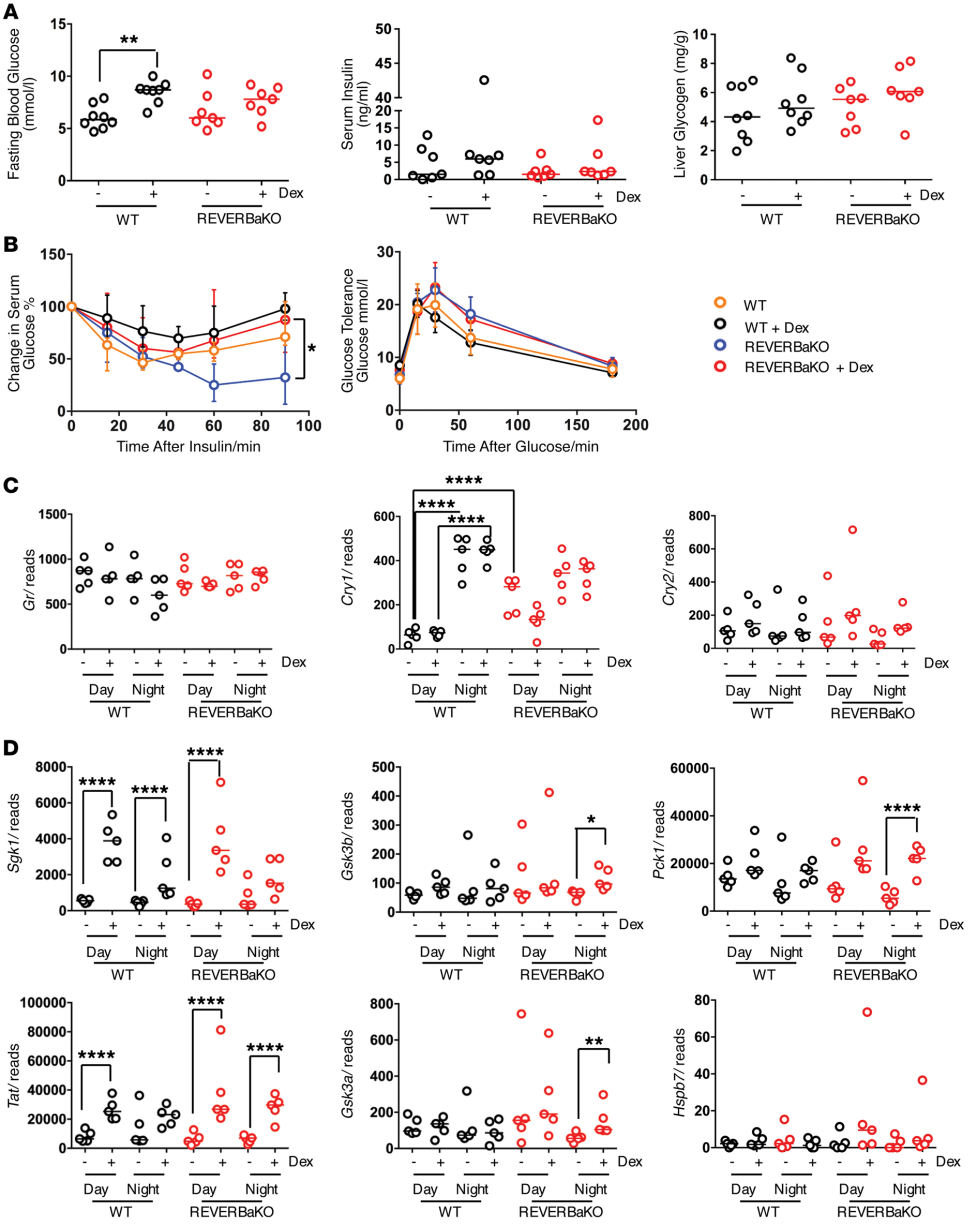
The effect of REVERBa on GC action is independent of cryptochromes. (**A**) C57BL/6 (WT) and REVERBaKO mice were given 1 mg/kg i.p. dex or vehicle at ZT6 every 48 hours for 8 weeks. Mice were assessed for effects on carbohydrate metabolism by fasting glucose (left), serum insulin (center), and liver glycogen content (right). (**B**) Mice were fasted for 4 hours and assessed for insulin tolerance (left) or fasted overnight for glucose tolerance (right) (*n* = 7–8). (**C**) Graphs show RNA-Seq reads for *Gr* (left), *Cry1* (center), and *Cry2* (right) target genes. (**D**) Graphs show RNA-Seq reads for known CRY1-regulated genes. Individual samples from RNA-Seq (*n* = 5) are all plotted with the median for each group. Individual values and median are shown for serum glucose, serum insulin, and liver glycogen. Mean values are shown for GTT and ITT. *q < 0.05, **q < 0.01, ****q < 0.0001, Fischer’s exact test adapted for negative binomial distribution (RNA-Seq). *P < 0.05, **P < 0.01, 2-way ANOVA (fasting glucose, GTT, serum insulin, liver glycogen, and ITT).

To investigate the impact of REVERBa on chronic GC regulation of carbohydrate metabolism, we first measured fasting glucose concentration after 8 weeks of alternate-day dosing with 1 mg/kg dex (29) (Figure 6A). Chronic GC led to a significant increase in circulating glucose only in WT animals. In contrast, the previously reported increase in insulin sensitivity characteristic of the REVERBaKO mice was lost in response to chronic GC treatment (Figure 6B), but there were no effects on glucose tolerance or liver glycogen content (Figure 6, A and B).

Previous work has shown that loss of CRY1 and CRY2 results in a gain of GR transactivation; we therefore measured the 2 CRY genes. In the REVERBaKO mice, daytime *Cry1* expression was higher than that in the WT mice, and there was no difference in nighttime *Cry1* expression between genotypes (Figure 6C). The increase in GC response seen at night in the REVERBaKO animals was therefore not attributable to CRY1, or CRY2. The subtle impact of REVERBa loss on GC regulation of carbohydrate metabolism is in contrast to the major changes reported to follow from *Cry1/2* double KO, where there was an amplification of the GC effect on gluconeogenesis (29). Therefore, the GC action phenotypes differed considerably between *Cry1/2* and *Reverba*. In our REVERBaKO mice, *Gsk3a*, *Gsk3b*, and the gluconeogenic genes *Pck1* and *Tat* all showed increased induction to GC at night, the time at which Cry1 expression is high, again suggesting a Cry-independent mechanism of action (Figure 6D).

**Figure 7:**
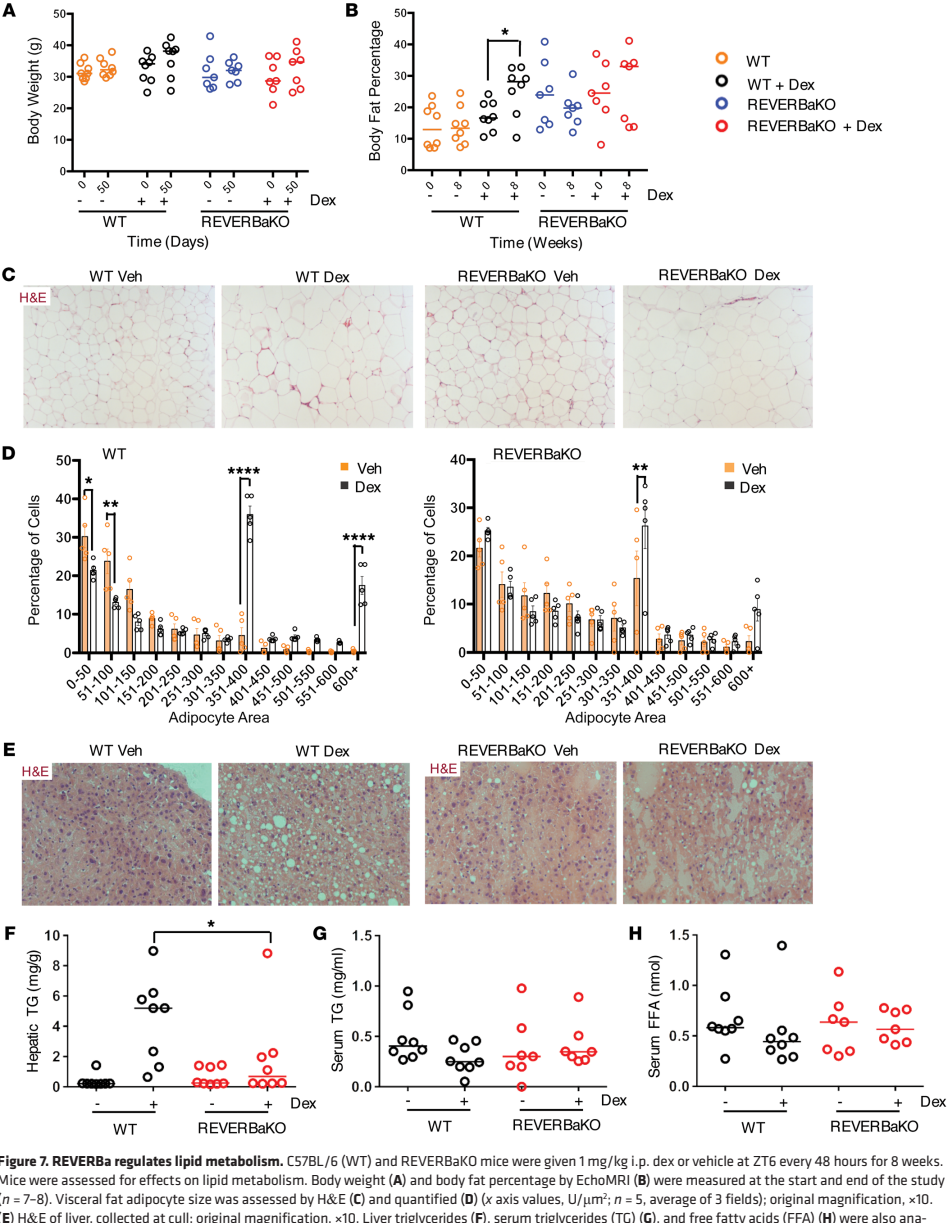
REVERBa regulates lipid metabolism. C57BL/6 (WT) and REVERBaKO mice were given 1 mg/kg i.p. dex or vehicle at ZT6 every 48 hours for 8 weeks. Mice were assessed for effects on lipid metabolism. Body weight (**A**) and body fat percentage by EchoMRI (**B**) were measured at the start and end of the study (*n* = 7–8). Visceral fat adipocyte size was assessed by H&E (**C**) and quantified (**D**) (*x* axis values, U/μm^2^; *n* = 5, average of 3 fields); original magnification, ×10. (**E**) H&E of liver, collected at cull; original magnification, ×10. Liver triglycerides (**F**), serum triglycerides (TG) (**G**), and free fatty acids (FFA) (**H**) were also analyzed (*n* = 8 liver, *n* = 7–8 serum). Graphs show data for individual animals with median; adipocyte area shows individual values and mean. Statistical analysis via 2-way ANOVA repeated measures (body weight and fat mass), 2-way ANOVA and 3-way ANOVA (adipocyte size), or Kruskal-Wallis test with a Dunn’s multiple comparison correction (hepatic triglycerides, serum triglycerides, and serum free fatty acids), *P < 0.05, **P < 0.01, ****P < 0.0001.

Chronic, low-dose GC treatment did not affect mouse body weight (Figure 7A). However, analysis of body composition by EchoMRI revealed a significant increase in body fat proportion in the GC-treated WT mice (Figure 7B). The REVERBaKO animals, in contrast, showed no significant change in fat mass in response to GC (Figure 7B). In agreement with this finding, dex treatment also increased adipocyte size and heterogeneity in WT mice, decreasing the fraction of small adipocytes and increasing the fraction of large adipocytes. This was seen to a lesser extent in the REVERBaKO mice (Figure 7, C and D, and Supplemental Figure 8), and analysis via 3-way ANOVA highlighted a significant interaction among treatment, genotype, and cell size (*P* < 0.0001). The catabolic actions of GC, with accumulation of adipose tissue, are characteristic of the changes seen in people treated with GC, and these changes impose a major limitation on therapeutic use of synthetic GC. It was intriguing that loss of REVERBa appeared to protect mice from this effect. Moreover, GC actions in the liver inhibit β-oxidation of fatty acids and ketogenesis, and promote synthesis of triglycerides, resulting in hepatosteatosis. *Reverba* deletion protected from GC-induced hepatic triglyceride accumulation, the most reliable quantitative measure of hepatosteatosis (Figure 7, E and F), with no effect on serum free fatty acids or triglycerides (Figure 7, G and H, and Supplemental Figure 9), suggesting that hepatic lipogenesis induced by GC is interrupted by loss of REVERBa.

### Emergence of HNF and epigenetic factors as important mediators of GR/REVERBa crosstalk in the liver.

**Figure 8:**
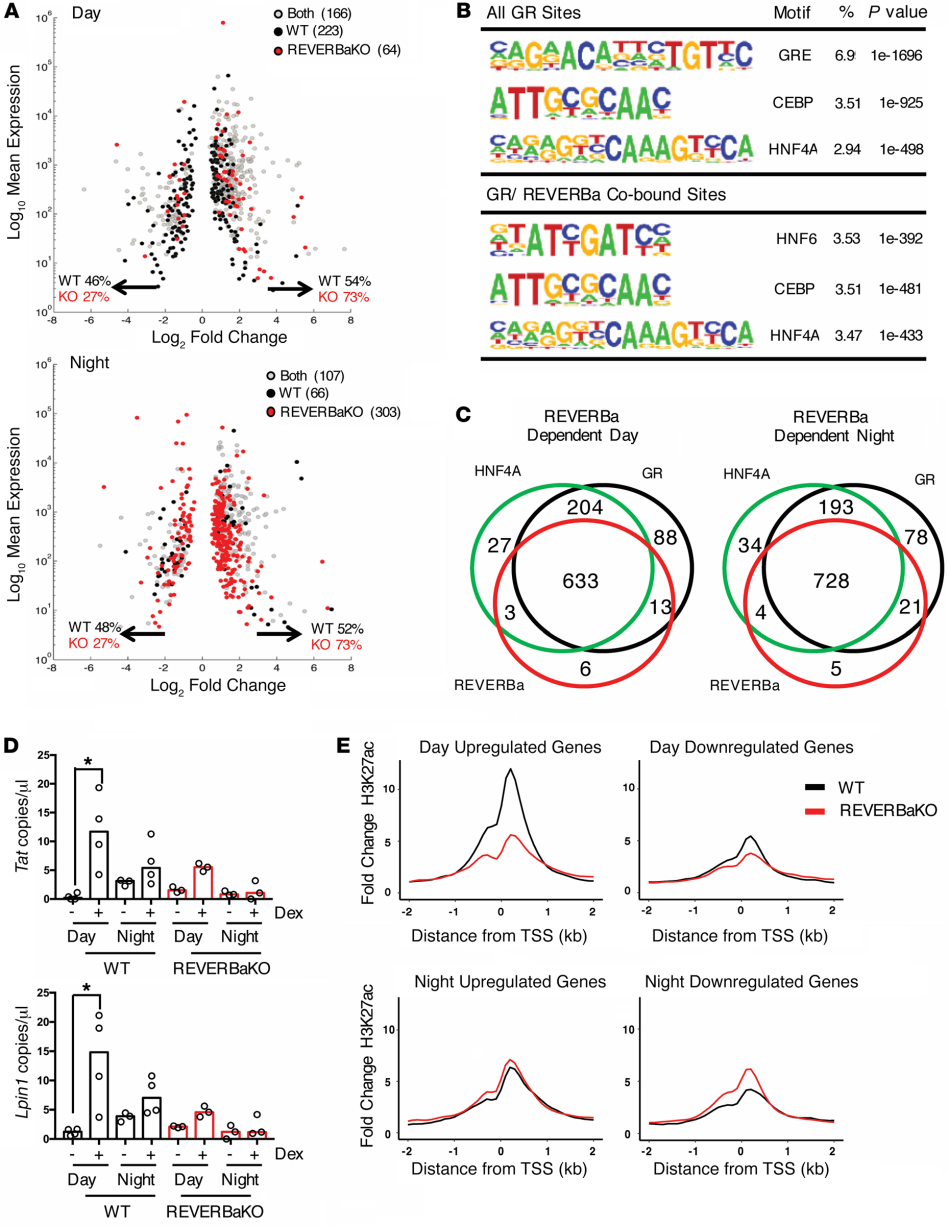
Rhythmicity of GC sensitivity in liver is determined by REVERBa and HNF transcription factors. (**A**) Base mean expression versus log_2_ fold change plots for GC-regulated genes in day and night show direction of gene regulation. (**B**) Summary of top-ranked motifs (by coverage [%], defined as fraction of observed/expected proportions taken from HOMER output) under all GR peaks or GR/REVERBa co-bound peaks (interpeak distance, <120 bp). (**C**) Overlay of GC-regulated genes with GR/REVERBa/HNF ChIP-Seq in the day and night. (**D**) ChIP-PCR for GR-REVERBa target genes in liver associated with carbohydrate (*Tat*) and lipid ontology (*Lpin1*) (*n* = 3–4); graphs show individual values and median. (**E**) C57BL/6 (WT) and REVERBaKO mice (male and female) were given 1 mg/kg i.p. dex or vehicle at ZT6 or ZT18 for 1 hour; livers were removed and fixed, and chromatin was immunoprecipitated for H3K27Ac (*n* = 2). Fold change in H3K27Ac coverage from prior to the transcription start site of genes upregulated or downregulated specifically during the day (top), and genes upregulated or downregulated specifically during the night (bottom) in WT and REVERBaKO mice identified from RNA-Seq in Figure 3. Statistical analysis via Mann-Whitney *U* test (ChIP-PCR), *P < 0.05.

In our studies, loss of *Reverba* resulted in 73% of GC-regulated genes being transactivated, in contrast to 54% in WT animals (Figure 8A). Motif analysis at sites of hepatic GR/REVERBa co-binding revealed enrichment for the hepatocyte-specific transcription factors HNF6 and HNF4A in motif analysis of all GR sites (Figure 8B and Supplemental Figures 5 and 6). There was a high degree of overlap between the ChIP-Seq–identified binding sites of the 3 relevant transcription factors, HNF4A, GR, and REVERBa, close to those GC target genes that were dependent on REVERBa. This was seen both for day and night-time GC-regulated genes (Figure 8C). For example, GC-regulated carbohydrate (*Irs1*, *Gck*) and fatty acid (*Dgat2, Lpin2*) metabolic genes show striking alignment of GR, REVERBa, and HNF4A binding (Supplemental Figure 7). Consistent with our findings, REVERBa was recently shown to be capable of binding DNA by tethering to HNF6 in a mechanism that does not require the REVERBa DNA binding domain (17), and HNF4A has previously been suggested as a GR pioneer factor in liver (22). REVERBa repressive action involves recruitment of NCOR and HDAC3 (17, 30), and indeed, many co-bound enhancers show time-of-day changes in histone H3K9Ac (Supplemental Figure 7), a mark regulated by HDAC3. These changes in histone modification are therefore predicted to be the result of productive engagement by the REVERBa repressive complex. Thus, our data support a mechanism of GR action that requires the HNF4A transcription factor, and time-of-day variation in chromatin accessibility.

To test directly the proposal that reduced GC transactivation by night, and in response to loss of REVERBa, resulted from impaired recruitment of GR and altered chromatin remodeling, we first examined GR binding at well-characterized enhancers related to the gluconeogenic gene *Tat* and the lipogenic gene *Lpin1* (Figure 8D). The nighttime loss of GR recruitment in WT mice was indeed also seen by day in the REVERBaKO mice, suggesting a shared mechanism of action and implicating REVERBa in the time-of-day change in GR function. We next performed ChIP-Seq analysis in WT and REVERBaKO mice for histone H3K27Ac, a robust marker for transcriptionally permissive and active chromatin (31) (Figure 8E). We examined genes regulated by GC by day and by night, using an approach similar to that previous applied to investigate circadian control of gene expression in the liver (28). The greatest differences between genotypes were seen in the transactivated genes, with a loss of daytime acetylation. There was little change in nighttime acetylation seen in the REVERBaKO animals, perhaps as nighttime is the nadir of REVERBa expression. This change is concordant with the observed changes in target gene response between genotypes, with WT mice having far more transactivated genes by day. This further supports a chromatin remodeling mechanism as the explanation for time-of-day and genotype differences in GC response, and again implicates REVERBa as a critical mediator of the time-of-day effect.

### HNF4A and HNF6 are required for time-of-day regulation of GR action in the liver.

**Figure 9:**
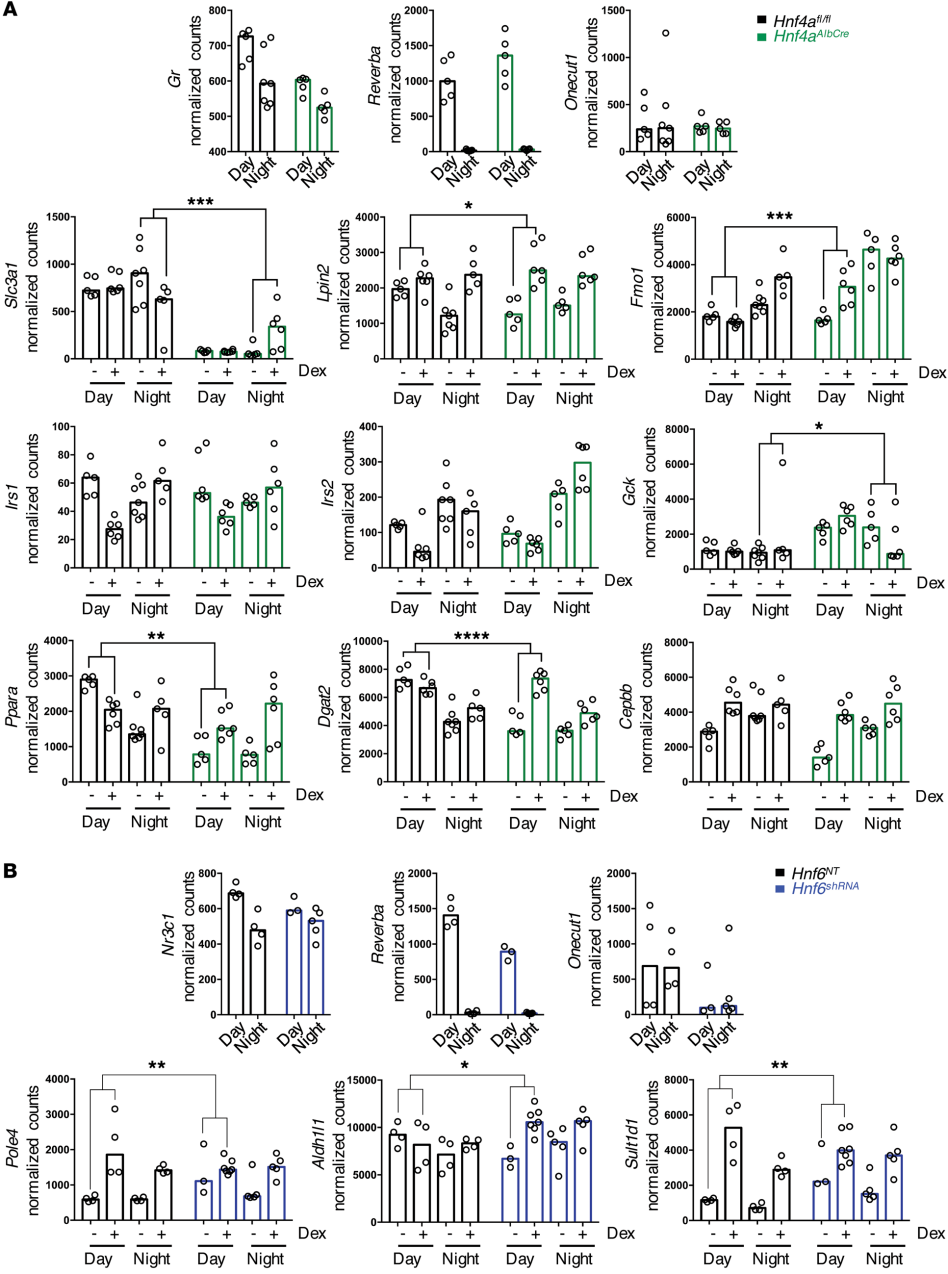
Hnf4a and Hnf6 selectively regulate rhythmic GC action. (**A**) *Hnf4a^AlbCre^* and *Hnf4a^fl/fl^* controls were treated with 1 mg/kg dex at ZT6 or ZT18 and culled 2 hours later. Livers were harvested, and RNA was analyzed via NanoString (*n* = 5–7 per group). (**B**) C57BL/6J mice were injected with shRNA against *Hnf6*. Mice were treated with 1 mg/kg dex at ZT6 or ZT18 and culled 2 hours later. Livers were harvested, and RNA was analyzed via NanoString (*n* = 3–7 per group). Genotype × treatment × time interactions were analyzed by limma; *q < 0.05, **q < 0.01, ***q < 0.001, ****q < 0.0001. Data shown for individual mice and median.

As 2 HNF transcription factors emerged as candidate mediators of the GR/REVERBa crosstalk in the liver, we sought direct evidence for their role in conferring time-of-day regulation to GR action. Initially we focused on HNF4A, using a liver-specific *Hnf4a*-KO mouse (*Hnf4a^fl/fl^*AlbCre) and Cre-negative littermates as control (32). The experimental design was the same as above, with GC administration timed to ZT6 or ZT18, followed by liver harvest 2 hours later and mRNA analysis. As older *Hnf4a^fl/fl^*AlbCre mice develop hepatic abnormalities, we used young mice (8 weeks of age), which showed no overt signs of illness, no differences in weight gain, and no gross hepatic abnormalities (33). We targeted 19 genes from the list of GR and REVERBa co-bound genes with a time-of-day difference in gene expression. Of these, 9 (47%) showed a genotype effect (Figure 9A, Supplemental Figure 12A, and Supplemental Table 12). We found no effect on GR gene expression or on *Reverba* expression (Figure 9A). We observed both gain and loss of temporal GR regulation (Figure 9A).

We also investigated HNF6, the other potential regulator identified in Figure 8. Here we used an AAV6-delivered shRNA approach. C57BL/6J mice were tail vein–injected with the AAV carrying either the targeting shRNA or a control sequence, and 3 weeks later the mice were treated with timed GC administration (ZT6 or ZT18), and mRNA analysis was conducted 2 hours later. We found that HNF6 knockdown was incomplete, with some variability between mice (Supplemental Figure 13, A and B), and therefore confined our analysis to livers where HNF6 protein expression was lower than the median level seen in the control mice (Supplemental Figure 13, A and B). Reduction of HNF6 expression also affected time-of-day GR action, but had no significant effect on either GR or *Reverba/b* gene expression (Supplemental Figure 12B). Unsurprisingly, we identified different genes affected by reduction in HNF6 compared with those seen in the *Hnf4a*-KO livers, as the 2 HNFs have different target sequence binding specificity (Figure 9B). We therefore define a complex GR regulatory network in liver where REVERBa carries critical timing information and the HNFs work cooperatively to confer liver specificity of effect.

## Discussion

Therapeutic use of GC in humans remains common, but is plagued by major off-target effects. GC use is a major risk factor for hepatosteatosis, a state leading to disruption of liver function, with resulting inflammation, fibrosis, and organ failure. There is renewed interest in timing of GC therapy, with nighttime release of prednisolone offering a small additional therapeutic effect (25). Our data suggest that a circadian, REVERBa-dependent mechanism exists that regulates GC regulation of hepatic lipid metabolism.

Our initial work simply tested the effect of timed administration of GC to intact WT mice. We observed clear organ-specific and time of day–specific differences in GC target gene expression. As previous work had elegantly shown a physical interaction between cryptochromes and the GR, with a functional effect on GC action in the liver, we studied the interaction between GR and various circadian clock proteins, including cryptochromes. In addition to cryptochromes, we also saw surprising co-binding between GR and the two REVERBs and confirmed this using coimmunoprecipitation, revealing REVERBa as a promising candidate mediating clock control of the GR, above and beyond the effects mediated by the cryptochromes.

To investigate the role of REVERBa, we repeated the acute GC dosing schedule in global REVERBaKO mice. There was a striking change, now with more genes regulated at night than in the day. Previous work identified a further potential point of crosstalk between GR and REVERBa, in that both nuclear receptors bind to the heat shock protein complex in the cytoplasm, and they exert reciprocal effects on nuclear localization (23). However, in our in vivo studies, we did not see a change in terms of number of target genes or amplitude of response, but rather a difference in the spectrum of target genes engaged by GR by time of day, and dependent on REVERBa.

The discovery that loss of REVERBa had such a major impact on GC regulation of both carbohydrate and lipid metabolic genes prompted us to examine the physiological impact in a chronic dosing study. In this protocol we used a low-dose dex administration schedule previously shown to reveal major changes in effect in the *Cry1/2* double-KO mice. The study was timed to ensure a duration of exogenous GC dosing similar to that in the previously published work (29). We observed small but significant increases in adiposity in littermate control mice in response to GC that were not seen in the REVERBaKO animals. The increased adiposity in littermate controls was accompanied by a small but significant increase in fasting glucose, but there was no difference in glucose tolerance tests between genotypes. Previous work had shown a marked increase in insulin sensitivity in the REVERBaKO mice, and we also observed this, by comparison to littermate controls. However, in response to chronic GC dosing, there was a significant loss of insulin sensitivity, but only in the REVERBaKO animals. Taken together we saw only minor changes in carbohydrate metabolic response to GC contingent on REVERBa expression, with loss of GC induction of adiposity in the REVERBaKO mice; and analysis of carbohydrate metabolic gene responses showed a number of changes but no consistent pattern (Supplemental Table 6). Analysis of lipid metabolism in the REVERBaKO mice showed minimal effects on serum triglycerides, but we saw striking protection from hepatic steatosis, which is typically a consequence of supraphysiological GC exposure, in the REVERBaKO mice. The absence of changes in serum lipid profile suggests a mechanism of action localized to the liver, with likely involvement of lipid synthetic genes (Supplemental Table 5).

The discovery that REVERBa serves as an additional mechanism linking the core circadian clockwork to GC action raised the question of mechanism. In our first studies, we saw major differences between metabolic (liver) and nonmetabolic (lung) tissue. In addition, we observed no impact of REVERBa loss on thymic atrophy or on liver inflammatory gene regulation by GC, suggesting very strong target gene and cell type specificity effects of the crosstalk. These observations prompted us to investigate the DNA sequences underlying peaks of GR/REVERBa co-binding in the liver. We saw a strong enrichment for the hepatic lineage-determining factors HNF4A and HNF6, which was interesting, as these factors had previously been found independently to be required for both GR and REVERBa recruitment to specific sites in the hepatocyte genome. Further analysis of the chromatin modification pattern at the co-bound sites uncovered time-of-day variation in the very histone mark most strongly associated with productive REVERBa recruitment, H3K9Ac. The coalignment of binding sequences for REVERBa, GR, and the sites of time-of-day variation in H3K9Ac is quite striking on genes shown to have time-dependent variation in GC response, including *Dgat2* and *Lpin2*. Therefore, we propose the existence of composite elements within energy metabolic genes, which become accessible in the liver under the influence of the HNFs, which serve as loci for crosstalk between GR and REVERBa. A prediction from this model is that recruitment of GR to its cognate enhancer elements in relation to these genes will be regulated by time of day, and also REVERBa status. Indeed, we did observe loss of GR recruitment to these sites at night in WT animals, when the GC response of these genes is lost, and a similar loss of GR recruitment in the REVERBaKO mice. Looking across all the GC-regulated genes, we saw a marked REVERBa effect, mainly on the H3K27 acetylation status of daytime GC-transactivated genes, which again fits with an underlying chromatin remodeling mechanism. Furthermore, using either liver-specific KO of *Hnf4a* or shRNA-mediated knockdown of *Hnf6*, we demonstrated that these nuclear receptors are required for gene-specific control of time-of-day GR responses at REVERBa-dependent sites.

Close cross-coupling between cellular clock machinery and GR is supported by overlapping roles in regulating (a) energy metabolism and immunity, (b) protein interactions between CRY and GR to affect carbohydrate metabolism, (c) and the potent clock-entraining activity of the GR, which transactivates *Per2* (22). To these we now add the physical interaction and functional cooperativity between GR and REVERBa. There has been limited application of timing information in GC therapy, with nighttime release of prednisolone offering a small additional therapeutic effect (25). Our studies suggest that timed administration of GC can be a powerful tool in targeting specific physiological programs, for example, by avoiding the detrimental metabolic actions of GCs in the liver but maintaining antiinflammatory activity.

## Methods

### Materials.

Anti–mouse GR antibody (catalog sc-1004) was purchased from Santa Cruz Biotechnology Inc.; anti–human GR antibody (catalog 24050-1-AP) was purchased from Proteintech. GSK6F05 anti-REVERBa was generated in collaboration with GlaxoSmithKline (34). ONECUT1 (HNF6) rabbit pAb (catalog 25137-1-AP, lot 00021988) and ACTB mouse mAb (catalog 0008-1, lot 0001084) were purchased from Proteintech. Dex, methyl-cyclodextrin, dextrose, and standard chemicals were purchased from Sigma-Aldrich.

### Cell lines.

Mycoplasma-free HEK293 cells were purchased from ATCC and maintained in DMEM (4,500 mg glucose/l, 110 mg sodium pyruvate/l, and l-glutamine, Sigma-Aldrich) with 10% v/v heat-inactivated FBS (Invitrogen) in a humidified atmosphere of 5% carbon dioxide at 37°C.

Primary bone marrow–derived macrophages were purified and cultured in growth media (DMEM containing 4,500 mg/l glucose, 110 mg/l sodium pyruvate, l-glutamine, and 10% heat-inactivated bovine serum) supplemented with 0.1 mg/ml M-CSF in a humidified atmosphere of 5% carbon dioxide at 37°C.

### Animals.

Experimentation was performed on mouse strains C57BL/6J (WT) from Harlan Blackthorn and global REVERBaKO mice and littermate controls imported from GlaxoSmithKline. There, the REVERBaKO mice were re-derived (from the original colony held by Ueli Schibler, Geneva, Switzerland) using in vitro fertilization procedures, with resulting heterozygous REVERBaKO animals subsequently backcrossed to a C57BL/6J background to over 98%, as confirmed by Marker-Assisted Accelerated Backcrossing (MAXBax, Charles River). Homozygous REVERBaKO and WT littermate controls were generated through heterozygous × heterozygous matings. Animals housed at GlaxoSmithKline were multiply housed in autoclaved Tecniplast GM500 IVC cages containing IPS Lignocel BK8/15 bedding with Datesand Paper Shavings nesting material, within a Tecniplast Smart Flow ventilation system. Animals were maintained at an ambient temperature of 20.5ºC to 23.5ºC and relative humidity of 39% to 61%, maintained on a 12-hour light/12-hour dark cycle, with free access to food (LabDiet Irradiated 5LF2 Maintenance Diet) and water. All mice (8–22 weeks old, male and female) were acclimatized in the biological services facility for 1 week before any procedures were undertaken. All procedures were performed in compliance with the Animals (Scientific Procedures) Act of 1986. While housed in Manchester, mice had free access to food (unless otherwise stated) and water and were multiply housed in a 12-hour light/12-hour dark cycle.

*Hnf4a^fl/fl^* and *Hnf4a^AlbCre^* mouse lines were described previously (32). Six- to 8-week-old male mice on a mixed SvJ129 and FVB background were used. Mice were housed in light- and temperature-controlled rooms and provided water and pelleted chow ad libitum.

### HNF6 shRNA targeting.

Twelve-week-old male C57BL/6J mice (Envigo) were used for HNF6-knockdown experiments. Animals were randomly allocated to treatment groups and coded. Samples were processed and decoded after analysis to limit any investigator bias. A custom adeno-associated viral vector against *Hnf6* (*Onecut1*) was generated. Mice were treated with 2E11 particles AAV6-EF1a-shmirOnecut1 (*n* = 20) or AAV6-EF1a-NTshmir (*n* = 16) (generated by Sirion Biotech) by tail vein injection. After 3 weeks, mice were used for experiments.

### Acute GC treatment.

Mice were treated at ZT6 (6 hours after lights on, 1:30 pm) or at ZT18 (6 hours after lights off, 1:30 am) with dex (1 mg/kg i.p.) or vehicle (1 mg/kg methylcyclodextrin i.p., *Hnf4a^fl/fl^* and *Hnf4a^AlbCre^* mice received saline vehicle) for either 2 or 4 hours before sacrifice by cervical dislocation.

### Chronic GC treatment.

Mice (*n* = 8) were treated at ZT6 (6 hours after lights on) with dex (1 mg/kg i.p.) or vehicle (saline i.p.) every 48 hours for 8 weeks before sacrifice by cervical dislocation. Two mice (one REVERBaKO vehicle, one REVERBaKO dex) were sacrificed prior to the end of the study for poor health. With the exception of hepatic triglyceride analysis, which is an early and robust GC response, all other samples from these animals were excluded from subsequent analysis.

### Glucose tolerance tests.

Mice were fasted overnight (12 hours) and injected at ZT6 with 2 g/kg dextrose. Blood glucose was measured over 3 hours (Aviva Accu-Chek). Glucose tolerance tests (GTTs) were performed prior to chronic treatment (week –1; WT *n* = 8 biological replicates, REVERBaKO *n* = 7 biological replicates).

### Insulin tolerance tests.

Mice were fasted for 4 hours and injected at ZT6 with 0.75 U/kg recombinant human insulin (Sigma-Aldrich, lot SLBM5131V). Blood glucose was measured over 90 minutes (Aviva Accu-Chek) (WT *n* = 8 biological replicates, REVERBaKO *n* = 7 biological replicates).

### Body weight and adiposity.

Mice were weighed every 48 hours between ZT3 and ZT5 and placed in an EchoMRI-900 (Echo Medical Systems) every 2 weeks between ZT6 and ZT9 for a total read time of between 140 and 160 seconds. An average of 3 readings were taken. Percent body fat was calculated from values determined by the EchoMRI (WT *n* = 8 biological replicates, REVERBaKO *n* = 7 biological replicates).

### Ex vivo LPS challenge.

Bones from REVERBaKO mice and littermate controls (males, aged 12–16 weeks) were collected and processed independently (*n* = 3). Primary cells were cultured in growth media supplemented with 0.1 mg/ml M-CSF for 7 days. On day 8, cell culture medium was replaced (without M-CSF), and cells were treated with vehicle or 100 nM dex for 1 hour and then 100 ng/ml LPS for a further 4 hours. Cells were lysed and processed for qRT-PCR.

### RNA-Seq.

Lung and liver were lysed and total RNA prepared using the SV Total RNA Isolation System (Promega). Quality and integrity of total RNA samples were assessed by a 2100 Bioanalyzer or a 2200 TapeStation (Agilent Technologies) according to the manufacturer’s instructions.

RNA-Seq libraries were generated using the TruSeq Stranded mRNA assay (Illumina) according to the manufacturer’s protocol, then paired-end sequenced (101 + 101 cycles, plus indices) on an Illumina HiSeq 2500 instrument. Demultiplexing of the output data (allowing one mismatch) and BCL-to-FastQ conversion were performed with CASAVA 1.8.3. FastQ files containing paired-end reads were quality checked with the FastQC tool, followed by Trimmomatic (35) and filtered reads aligned to the GRCm38.71 (mm10) assembly of the mouse genome using TopHat 2.0.11 (36). Mapped reads to genes were counted, for Ensembl annotation GRCm38.71, using HTSeq-count (v0.5.4p5) (37), with default quality score and with options “stranded=reverse” and “intersection_nonempty.”

DESeq2 (38) was used to perform normalization and pairwise comparisons. Differentially expressed (DE) genes were reported for *q* ≤ 0.05 and fold change of 2 (Figure 1) or *q* ≤ 0.1 (Figure 3) and were taken forward for downstream analysis. The online bioinformatics tools webgestalt (http://www.webgestalt.org/) and Enrichr (http://amp.pharm.mssm.edu/Enrichr/) were used for enrichment analysis of the DE genes. Sequencing datasets were deposited in Array Express under the accession numbers E-MTAB-7020, E-MTAB-7017, and E-MTAB-6994.

### qRT-PCR.

Lung, liver, and macrophages were lysed, and total RNA was prepared using the SV Total RNA Isolation System. Total RNA was reverse transcribed to cDNA using a High Capacity RNA-to-cDNA Kit (Applied Biosystems) and subjected to qRT-PCR using SYBR Green (KAPA Biosystems) detection in a StepOnePlus Real-Time PCR System (Applied Biosystems). All samples were analyzed in duplicate. Expression levels were calculated using the δδCT method, normalizing to β-actin control (lung and liver, *n* = 4 biological replicates; macrophages, *n* = 3 biological replicates).

### Mitochondrial genome quantification.

Livers and lungs were homogenized, DNA was extracted using TRIzol (Invitrogen), and relative mitochondrial DNA (ND1) was quantified using qRT-PCR (Applied Biosystems). Expression levels were calculated using the δδCT method, normalizing to the nuclear genome (GAPDH). Primer sequences are described in Supplemental Table 7 (*n* = 10 for all lung treatment groups; *n* = 10 vehicle liver ZT6, *n* = 9 dex liver ZT6 and vehicle liver ZT18, and *n* = 7 dex liver ZT18).

### Nanostring RNA analysis.

Total RNA was extracted from flash-frozen liver tissue (ReliaPrep RNA Tissue Miniprep System, Promega). Gene expression in 100 ng RNA was quantified using NanoString nCounter technology, using a custom codeset of probes (Supplemental Tables 8–12). mRNA counts were normalized to the housekeeping genes Actb and B2m using nSolver software (NanoString). Gene expression analysis was performed on log_2_-transformed normalized counts using the Bioconductor package limma (39), with an FDR (Benjamini-Hochberg) threshold of 0.05.

### Transcription factor ChIP-Seq analysis.

GR ChIP-Seq data from GEO GSE46047 (9) were downloaded, and FastQ files were mapped to the mm10 genome using Bowtie1 (40) with default options except the “−m1” option, keeping only uniquely mapped reads. Peaks were called using MACS 2.1.0 (41) with default parameters, except the “–nolambda” option. To associate identified peaks to mm10 annotated genes, HOMER (42) (annotatePeaks.pl) was used, with default options.

### Co-binding analysis.

ChIP-Seq datasets (BED files for mm9 assembly) for GR (GR 6:00 am/6:00 pm; GSE59764 (12) and core clock transcription factors (GSE3986, ref. 28, and GSE34020, ref. 16) were downloaded from GEO and lifted to the mm10 assembly using the UCSC liftOver tool (https://genome.ucsc.edu/cgi-bin/hgLiftOver). For each of the clock transcription factors, histograms were drawn for summit-to-summit distances between each GR peak and its nearest clock transcription factor peak, as described previously (43).

### Motif analysis.

Regions from the GR BED file with a REVERBa peak within 120 bp were extracted, and HOMER (findMotifsGenome.pl) was used to find enriched de novo and known motifs in these co-bound regions, as well as locations of top motifs. Motifs were built using the options “-len 8,12,15,” and “-size 200,*”* while when finding locations of the selected motifs, the option “-size given*”* was used. Output motifs were ranked based on %Ratio (observed/expected frequencies). Duplicate motifs, or motifs with %Ratio <1.5 or total coverage <5%, were removed.

### ChIP.

ChIP was performed using the ChIP-IT High Sensitivity kit (Active Motif) according to manufacturer’s instructions. A TissueRuptor (QIAGEN) was used for 45 seconds at maximum speed to disrupt liver tissue, followed by Dounce homogenizing for 70 strokes to release cell nuclei. Nuclear sonication was conducted with an EpiShear Probe Sonicator (Active Motif, 3.5-mm probe) for 8 rounds of 2 minutes per sample, at 37% amplitude. 15 μg sheared chromatin was incubated with anti-H3K27Ac antibody (Active Motif, lot 31814008) or an anti-GR antibody cocktail (Proteintech 24050-1-A, lot 00044414, and Cell Signaling Technology, D8H2, lot 2). For GR ChIP, 30 ng spike-in *Drosophila melanogaster* chromatin (Active Motif) plus an antibody to the *Drosophila*-specific histone variant H2Av (Active Motif Spike-in Antibody 61686; lot 34216004) (44) were include in the IP reaction. Antibody was pulled down with magnetic protein G agarose microspheres (ReSyn Biosciences). After elution and de-crosslinking, ChIP DNA was purified, as per the kit instructions.

### H3K27ac ChIP-Seq.

5–10 ng ChIP DNA was submitted to the University of Manchester Genomics Facility for library preparation and paired-end sequencing on the Illumina HiSeq 4000 platform. Reads were trimmed and aligned to the mm10 genome using Bowtie 2 (45). Reads were counted (in 100-bp windows) for the lists of dex up-/downregulated genes (as identified from RNA-Seq in Figure 3) in both C57BL/6 (WT) and REVERBaKO mice using BEDTools (46). The sites are redefined as 2 kb either side of the TSS to make them symmetrical. Regions that overlap with ChIP-Seq blacklisted regions were filtered out. The data are presented as fold change compared with average read count in the 1 kb preceding the site. The values plotted are averages over the total number of sites.

### GR ChIP-ddPCR.

2 μl ChIP DNA was added to each PCR reaction comprising QX200 ddPCR EvaGreen Supermix (Bio-Rad) and primers listed in Supplemental Table 7. To normalize ddPCR results, one spike-in value (as copies per microliter of the PCR reaction) was chosen as the reference value. For each other sample, the reference spike-in value was divided by that sample’s spike-in value, to create a normalization factor. Values for the targets of interest (also as copies per microliter of the PCR reaction) were then multiplied by this normalization factor, as per the manufacturer’s (Active Motif) instructions.

### Histology.

Lungs were immediately infused with 1 ml of 4% paraformaldehyde then submerged in 4% paraformaldehyde overnight. Livers and visceral adipose were submerged in 4% paraformaldehyde overnight. Tissues were embedded into paraffin blocks and cut into 5-μm sections (Leica RM2255 Microtome).

For antibody staining, sections were dewaxed using xylene, rehydrated through an alcohol series, then water and endogenous peroxidase quenched with 0.003% v/v H_2_O_2_. Samples were washed with PBS, antigen retrieved (10 mM citric acid, pH 6) for 20 minutes, and then washed again in PBS. After avidin/biotin blocking (Vector Laboratories), sections were incubated with primary antibody (GR M20, Santa Cruz Biotechnology Inc., 1:200 in PBS/0.1% v/v Triton X-100/3% goat serum) overnight at 4°C. Sections were washed with PBS, incubated with biotinylated anti-rabbit (Vector Laboratories, 1:800 in PBS/0.1% v/v Triton X-100) for 2 hours at 4°C, washed again with PBS, and then incubated with streptavidin-conjugated horseradish peroxidase (1:200 in PBS) for 1 hour at 4°C. After final washes in PBS, color detection was performed using DAB (Vector Laboratories), and nuclei were counterstained with toluidine blue. Sections were then dehydrated through an alcohol series, then xylene, and mounted using Entallan (Merck).

For the H&E staining, paraffin-embedded adipose or liver sections were rehydrated through an alcohol series and brought to distilled water. Nuclei were stained with hematoxylin (2 minutes); rinsed in tap water; stained with eosin (2 minutes); rinsed with tap water; dehydrated through an alcohol series, then xylene; and mounted using Entallan.

All images were acquired on an Axio Imager.A1 (Zeiss) microscope using either a ×10 or ×20 Zeiss EC Plan-Neofluar objective, using AxioCam MRc (Zeiss). Raw images were visualized using AxioVision Rel. 4.7 (Zeiss), processed, and quantified using the NIH ImageJ Adipocytes Tool (http://dev.mri.cnrs.fr/projects/imagej-macros/wiki/Adipocytes_Tool).

### Metabolic profiling.

Quantification of ATP, ADP, AMP, NAD, NADP, and NADPH from flash-frozen liver was performed by the Metabolomics Innovation Centre (TMIC) at the University of Alberta.

### Immunoprecipitation.

HEK293 cells were transfected with 1 μg halo-tagged REVERBa and/or 1 μg halo-tagged GR using polyethylenimine (PEI) (3:1 v/w ratio) and left overnight. Cells were transferred to media containing charcoal-stripped FBS (Invitrogen) 4 hours before treatment with dex (100 nM) or DMSO for 1 hour, then lysed (150 mM NaCl, 20 mM Tris-HCl, 10% glycerol, 1% Triton X-100, 1 mM PMSF, 10 mM *N*-ethylmaleimide [NEM], PhosSTOP, cOmplete EDTA-free protease inhibitor cocktail [MilliporeSigma]) on ice, and cell debris was cleared by centrifugation. 1 μg anti-REVERBa antibody (mouse monoclonal GSK6F05, or 1 μg mouse IgG) or 1 μg anti GR antibody (rabbit polyclonal, Proteintech, or 1 μg rabbit IgG) was incubated with protein lysates for 1 hour, and antibody complexes were captured using magnetic beads (Surebeads protein G magnetic beads, Bio-Rad; MagReSyn Protein A, ReSyn Biosciences) for 45 minutes at 4°C. Beads were washed with lysis buffer, then boiled in SDS loading dye.

### Immunoblotting.

Livers were lysed (150 mM NaCl, 20 mM Tris-HCl, 10% glycerol, 1% Triton X-100, 1 mM PMSF, 10 mM NEM, PhosSTOP, cOmplete EDTA-free protease inhibitor cocktail) and protein quantified using Bradford Assay (Bio-Rad). Samples were electrophoresed on Mini PROTEAN TGX Precast Gels 4-15% (Bio-Rad) and transferred to 0.2-μm Protran nitrocellulose membranes. Membranes were blocked (5% skim milk powder in TBST, 50 mM Tris, 150 mM NaCl, pH 7.6, 0.1% Tween-20) for 1 hour at room temperature and then incubated with mouse monoclonal GSK6F05 anti-REVERBa, rabbit polyclonal anti-GR (Proteintech), mouse monoclonal anti-ACTB (Proteintech), or rabbit polyclonal anti-ONECUT1 (HNF6) (Proteintech) antibodies overnight at 4°C. Membranes were washed in TBST and secondary HRP-linked antibodies (GE Healthcare), or Dylight-680/800 secondary antibodies (Cell Signaling Technology) were incubated for 1 hour. After washes in TBST, immunoreactive bands were detected using SuperSignal West Dura (Thermo Fisher Scientific), and chemiluminescence was visualized on Kodak BioMax MR or XAR Film or LI-COR Odyssey CLx.

### Corticosterone measurements.

Blood was left to clot for 30 minutes and centrifuged for 10 minutes at 1,000 *g*, and serum was collected. Serum was diluted 1:40, and duplicate samples were run alongside a standard curve of corticosterone (range 32–27,000 pg/ml) according to the manufacturer’s instructions (Corticosterone ELISA kit, catalog ADI-900-097, Enzo).

### Insulin measurements.

Blood was left to clot for 30 minutes and centrifuged for 10 minutes at 1,000 *g*, and serum was collected. Serum was diluted 1:2, and samples were analyzed alongside standard a curve of insulin (range 37–150,000 pg/ml) according to the manufacturer’s instructions (MILLIPLEX MAP Mouse Bone Magnetic Bead Panel, catalog MBNMAG, Millipore) using the Bioplex 200 system (Bio-Rad) (WT *n* = 8, REVERBaKO *n* = 7 biological replicates).

### Liver glycogen measurements.

Liver homogenates (10 mg/100 μl) were boiled for 5 minutes and cleared by centrifugation at 13,000 *g* for 10 minutes. Single measurements of 0.5 and 0.1 μl sample/well were run alongside a standard curve of glycogen (range 0.2–2 μg) according to the manufacturer’s instructions (Glycogen Assay Kit, catalog MAK016, Sigma-Aldrich) (WT *n* = 8, REVERBaKO *n* = 7 biological replicates).

### Triglyceride and free fatty acid measurements.

Liver homogenates (10 mg/100 μl) and serum were isolated. Undiluted serum samples and liver homogenates (diluted 1:2) were run in duplicate alongside a standard curve of glycerol (range 0.037–2.5 mg/ml, triglyceride assay) or palmitic acid (range 0.2–1 nmol, free fatty acid assay) according to the manufacturer’s instructions (Serum Triglyceride Determination Kit, catalog TR0100; Free Fatty Acid Quantitation Kit, catalog MAK044; Sigma-Aldrich) (WT *n* = 8, REVERBaKO *n* = 8 biological replicates for liver; WT *n* = 8, REVERBaKO *n* = 7 for serum analysis.

### Statistics.

Standard statistical tests were completed using GraphPad Prism, and data are presented as group means with SD, SEM, or as individual data points with median (as indicated in the figure legends). D’Agostino-Pearson test was used to determine whether data were normally distributed, and parametric or nonparametric tests used accordingly, determined by pairwise or multiple comparisons. Data were analyzed by 2-tailed Student’s *t* test or 2-tailed Mann-Whitney *U* test, 1-way ANOVA with Holm-Šidák post hoc analysis, Kruskal-Wallis with Dunn’s multiple comparisons test, 2-way ANOVA with the Tukey’s post hoc test, or 3-way ANOVA with the Tukey’s post hoc test.

### Study approval.

All animal studies were approved by the University of Manchester and performed in accordance with the Animals (Scientific Procedures) Act (1986) (United Kingdom) or in accordance with the Association for Assessment and Accreditation of Laboratory Animal Care International guidelines and approved by the National Cancer Institute Animal Care and Use Committee (USA).

## Author contributions

GC, LCM, and DWR conceived the project. GC, DK, and LH completed in vivo studies. GC and LH processed and analyzed tissue and primary data. GC, MI, PW, IJD, and RM performed statistical analysis. MI and IJD analyzed RNA-Seq and ChIP-Seq data, performed co-binding and motif analysis, and modeled time series data. PW analyzed RNA-Seq data. RMV, NB, MP, ZZ, GK, LMI, MB, TMP, and DAB assisted with in vivo studies. DK, CB and FG provided mice. LH completed GR ChIP-ddPCR. GC, AJT, and JLW completed biochemical assays. MP completed coimmunoprecipitation studies. ZZ performed ex vivo LPS challenge in macrophages. DRS and DAD generated the REVERBa mAb. ASIL, DAB, MR, LCM, and DWR supervised the project. GC, DAB, MR, LCM, and DWR wrote the manuscript. GC and LCM prepared the figures. All authors edited the manuscript.

## Supplementary Material

Supplemental data

## Version 1 09/04/2018

Electronic publication

## Version 2 10/01/2018

Print issue publication
