# Supplementary material for: REVERBa couples the circadian clock to hepatic glucocorticoid action
Source: J Clin Invest. 2018 Sep 4;128(10):4454–71. doi: 10.1172/JCI96138 (PMC6160001; doi:10.1172/JCI96138)
Supplement: Supplemental data [file jci-128-96138-s155.pdf]

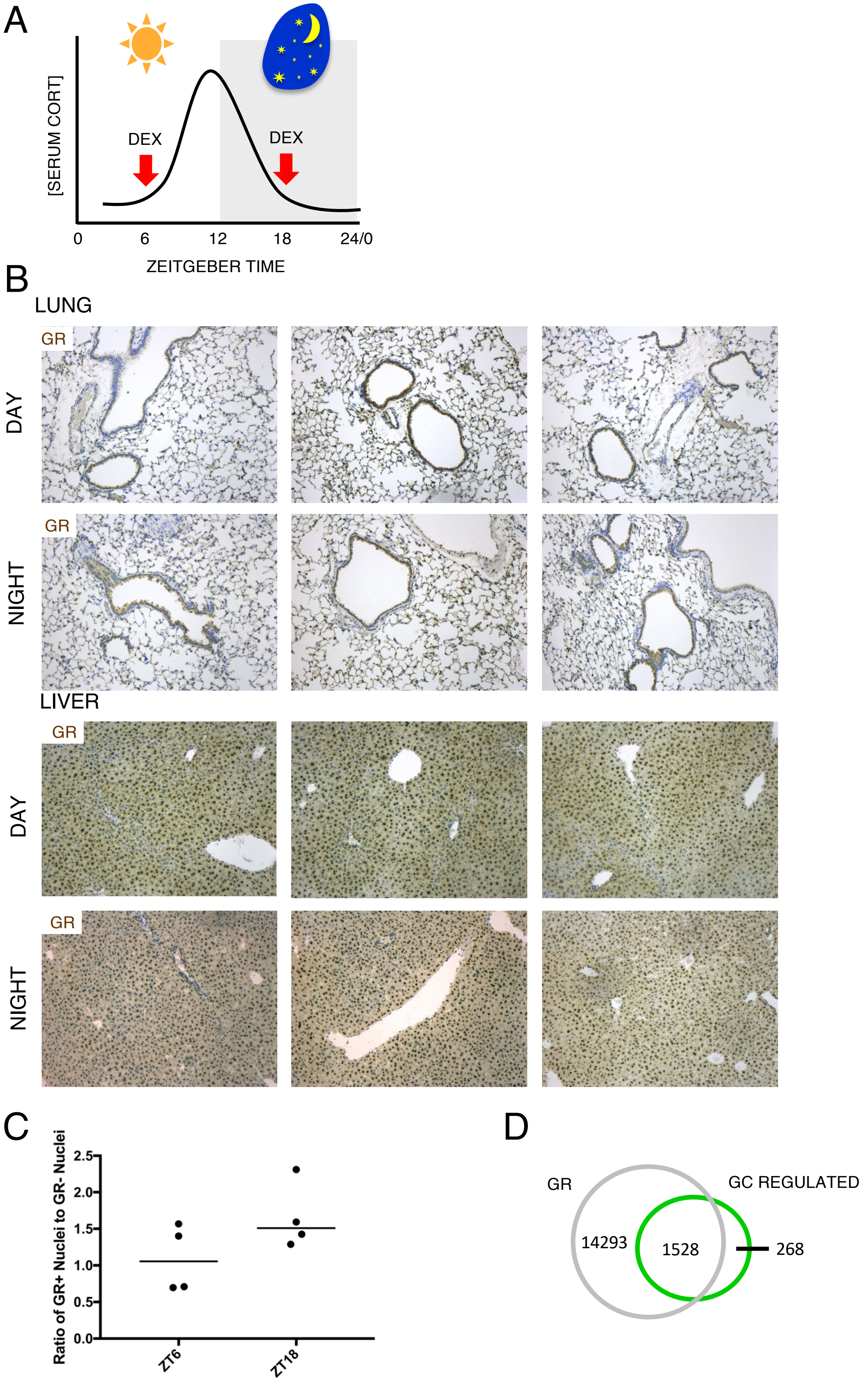

Figure S1. GR expression does not alter throughout the day in lung or liver. C57BL/6 mice were given vehicle or 1mg/kg I.P. dexamethasone at ZT6 (1pm, day) or ZT18 (1am, night), culled 2 hours later and lung and liver analysed by RNA-seq (**A**) Lung and liver were analysed by immunohistochemistry for GR expression (brown) and nuclei counterstained with toluidine blue. Three examples are shown. 10x magnification (**B**). GR nuclear localisation in liver was quantified using ImageJ, 1 field from 4 mice were analysed (**C**). GC regulated genes in the liver were compared to ChIP-seq annotated genes bound by GR in mouse liver (**D**).Statistical analysis by Mann-Whitney test.

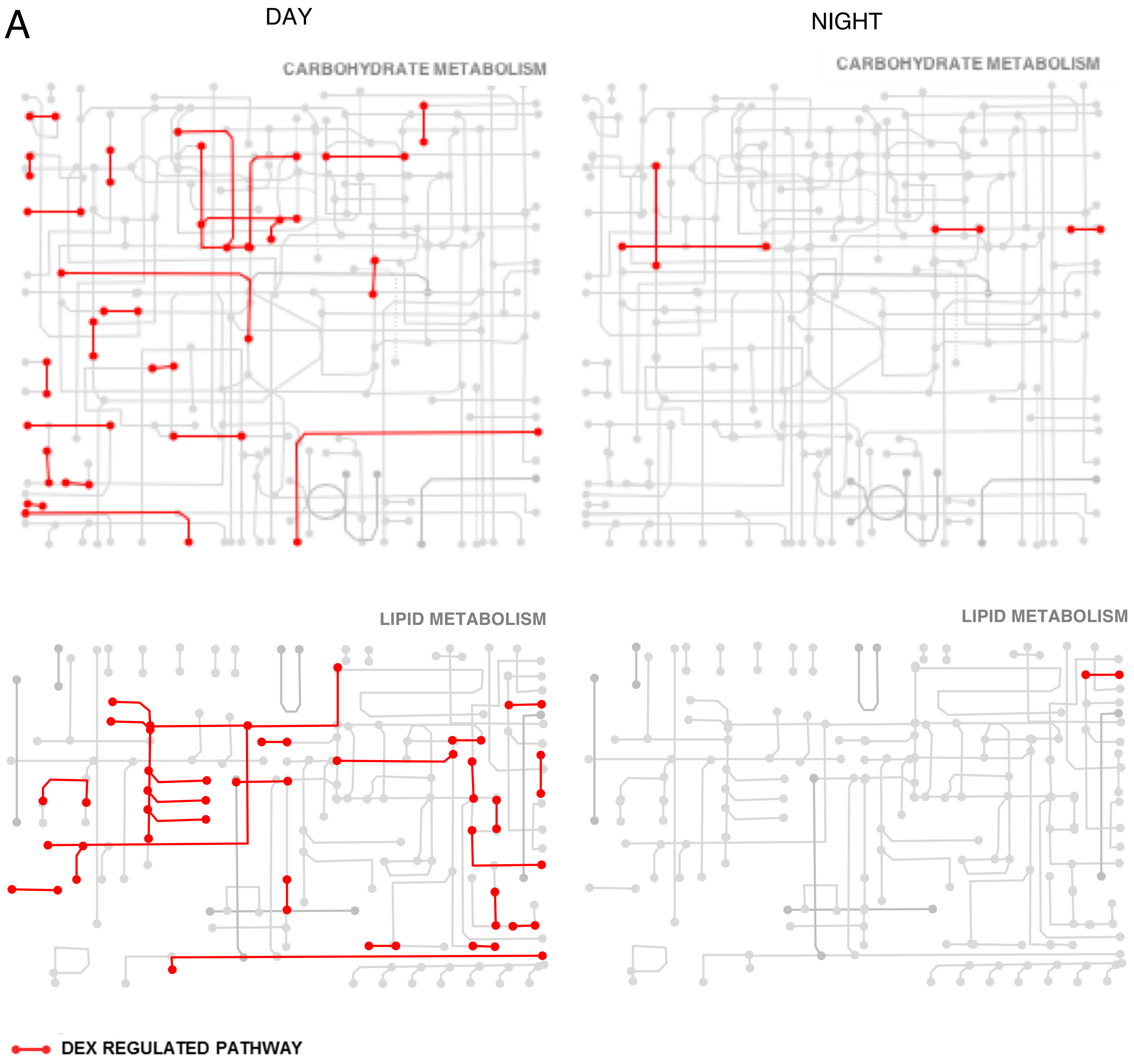

Figure S2. Day specific GC regulated genes in liver control key metabolic pathways. Schematic summarizing KEGG Pathway analysis of GC regulated genes in liver. GC regulated pathways are shown in red.

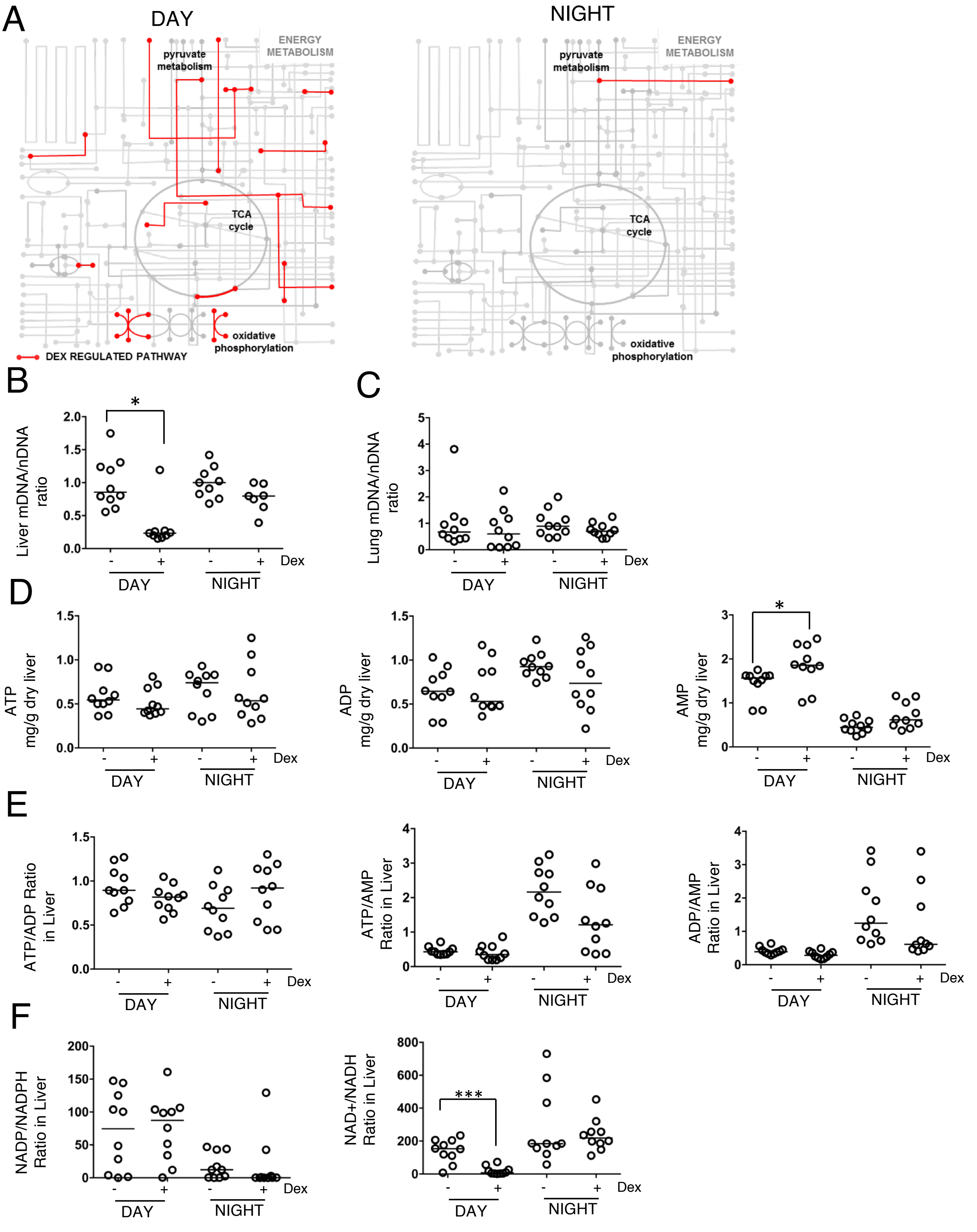

Figure S3. GC reduces mitochondrial number in the liver only in the day. Schematic summarizing KEGG Pathway analysis of GC regulated genes in liver related to mitochondrial metabolism. GC regulated pathways are shown in red (**A**) C57BL/6 mice were treated with 1mg/kg I.P. dexamethasone at either ZT6 or ZT18 and culled 4 hours later. Relative mitochondrial DNA in liver (N=7-10) (**B**) and lung (N=10) (**C**), were normalised to vehicle. AMP, ADP, ATP (**D**) and NADP, NADPH, NAD+ and NADH (**E**) were measured via HPLC in dry mouse liver (N=10). Ratios of NADP to NADPH and NAD+ to NADH (**F**) were calculated. Data shown as median. Statistical analysis of GC treatment via one-way ANOVA with a Holm-Sidak multiple comparisons correction (mitochondrial quantification AMP, ADP and ATP) or one-way ANOVA, followed by a t-test NADP, NADPH, NAD+ NADP/NADPH ratio and NAD+/NADH ratio), $p < 0.05$ * $p < 0.001$ **, $p<0.0001$ ***

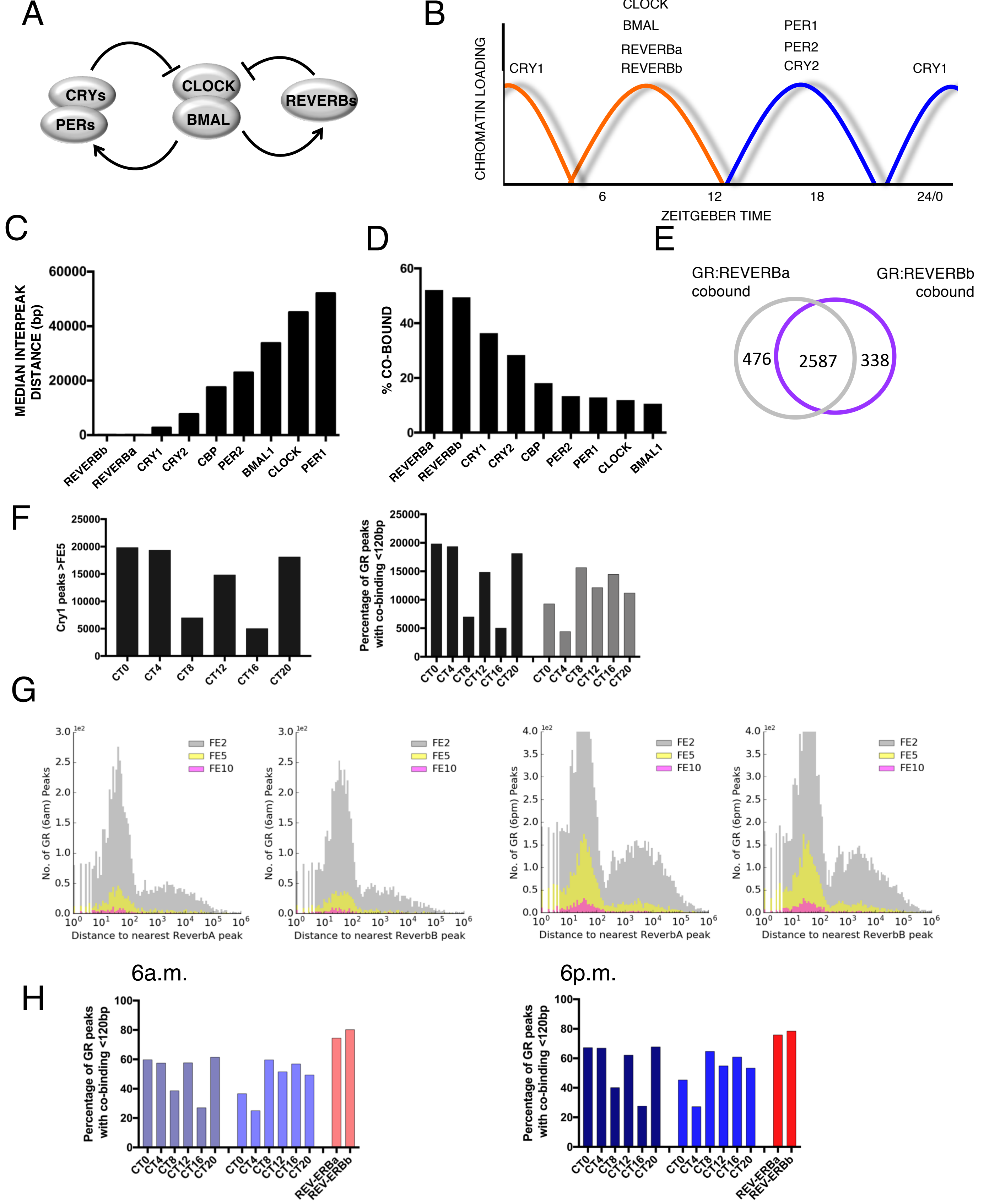

Figure S4. GR binding shows minimal overlap with binding of some core clock transcription factors. Cartoon of core clock feedback loop (**A**) and representation of data from Koike et al (**B**). ChIP-seq data for GR and core clock transcription factors was compared to determine proximity of binding. Histograms depict the number of GR binding peaks against distance from closest transcription factor summit using three stringencies (fold enrichment (FE) scores). Median interpeak distances for the highest stringency (FE30) are plotted in order of ranking (**C**). The percentage of GR co-binding with each transcription factor is also plotted (**D**). Genes co-bound by GR and REV-ERBa or REV-ERBb (**E**). Enrichment of CRY1 peaks at 6 time points, and the co-binding percentage of CRY1 or CRY2 with GR cistrome taken from 6 a.m. and 6 p.m. (**F**). REV-ERBa and REV-ERBb co-binding with GR at either 6 a.m. (**G, left**) or 6 p.m. (**G, right**). CRY1 and CRY2 peaks throughout a circadian cycle are shown, and co-binding between GR cistromes at either 6 a.m. (**H, left**) or 6 p.m. (**H, right**) was analysed.

ALL GR BINDING SITES, %RATIO >1.5, TARGET COVERAGE >5%

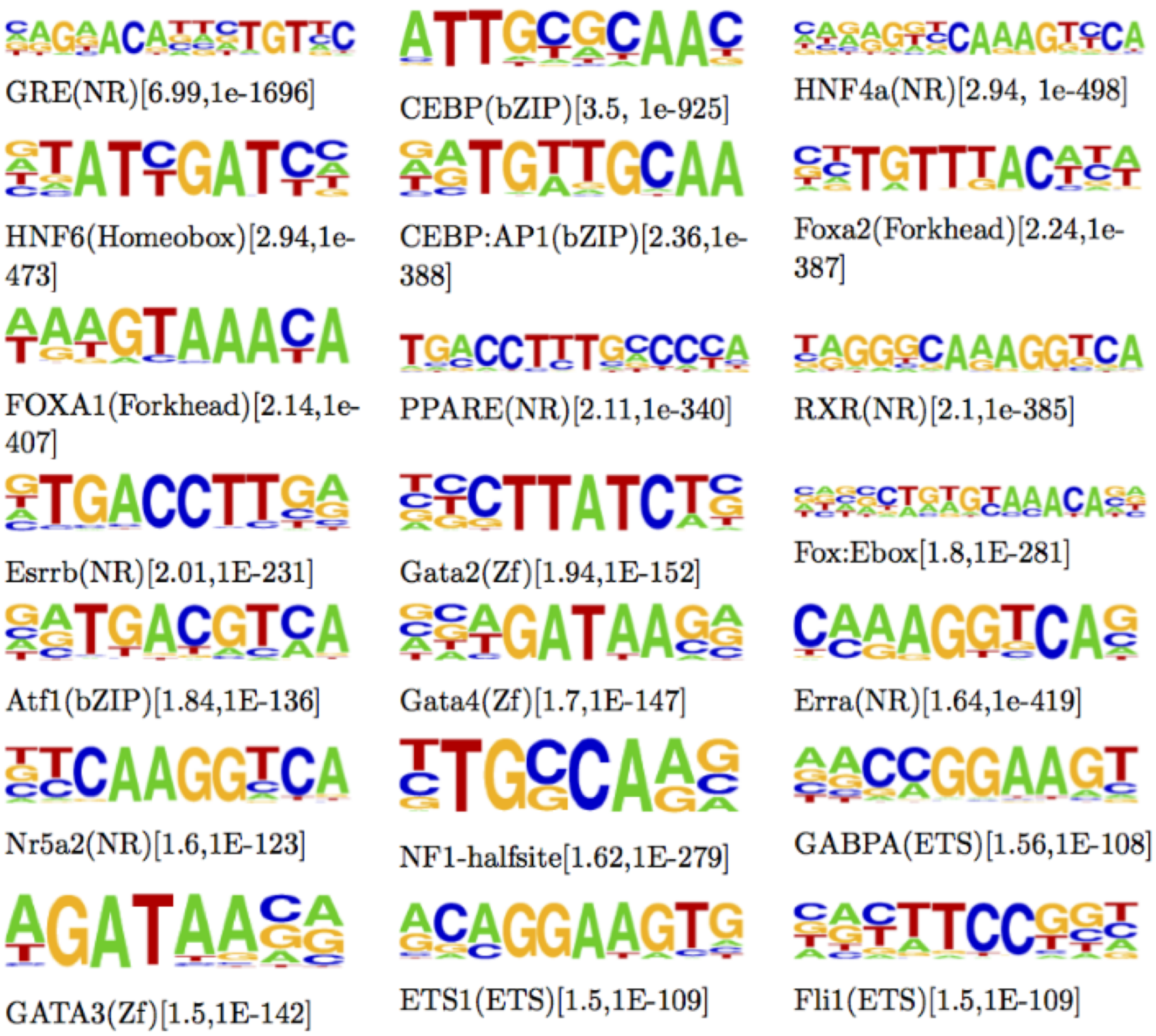

Figure S5. Analysis of all GR binding sites shows enrichment of GREs. Motif analysis for all GR binding sites. Observed/Expected ratios, and p-values are indicated for each motif.

COMMON GR/REVERBα BINDING SITES, %RATIO >1.5, TARGET COVERAGE >5%

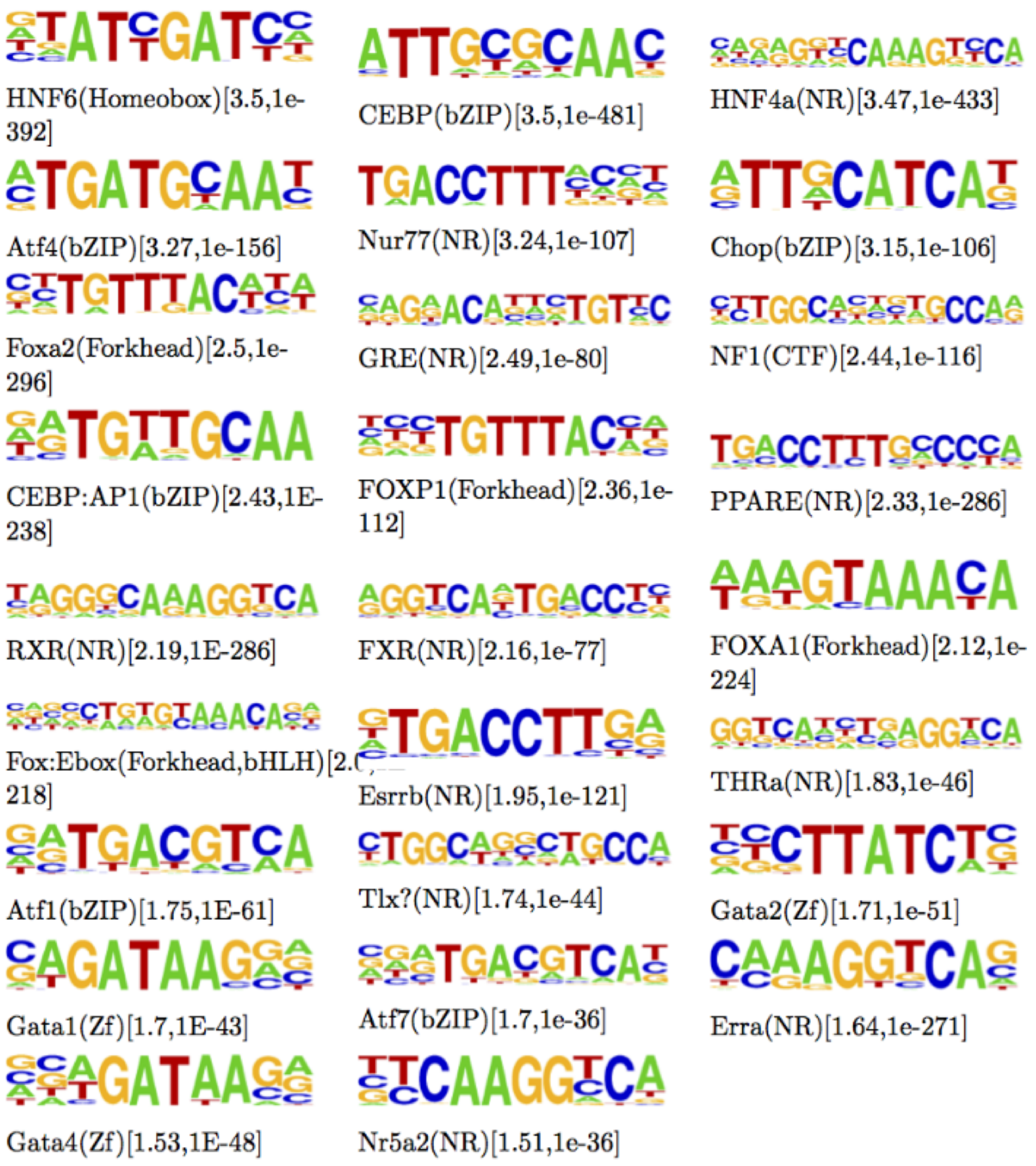

Figure S6. GR-REV-ERBa co-bound regions are enriched for HNF motifs. Motif analysis for all GR-REV-ERBa co-bound sites (where binding occurs within 120bp). Observed/Expected ratios, and p-values are indicated for each motif.

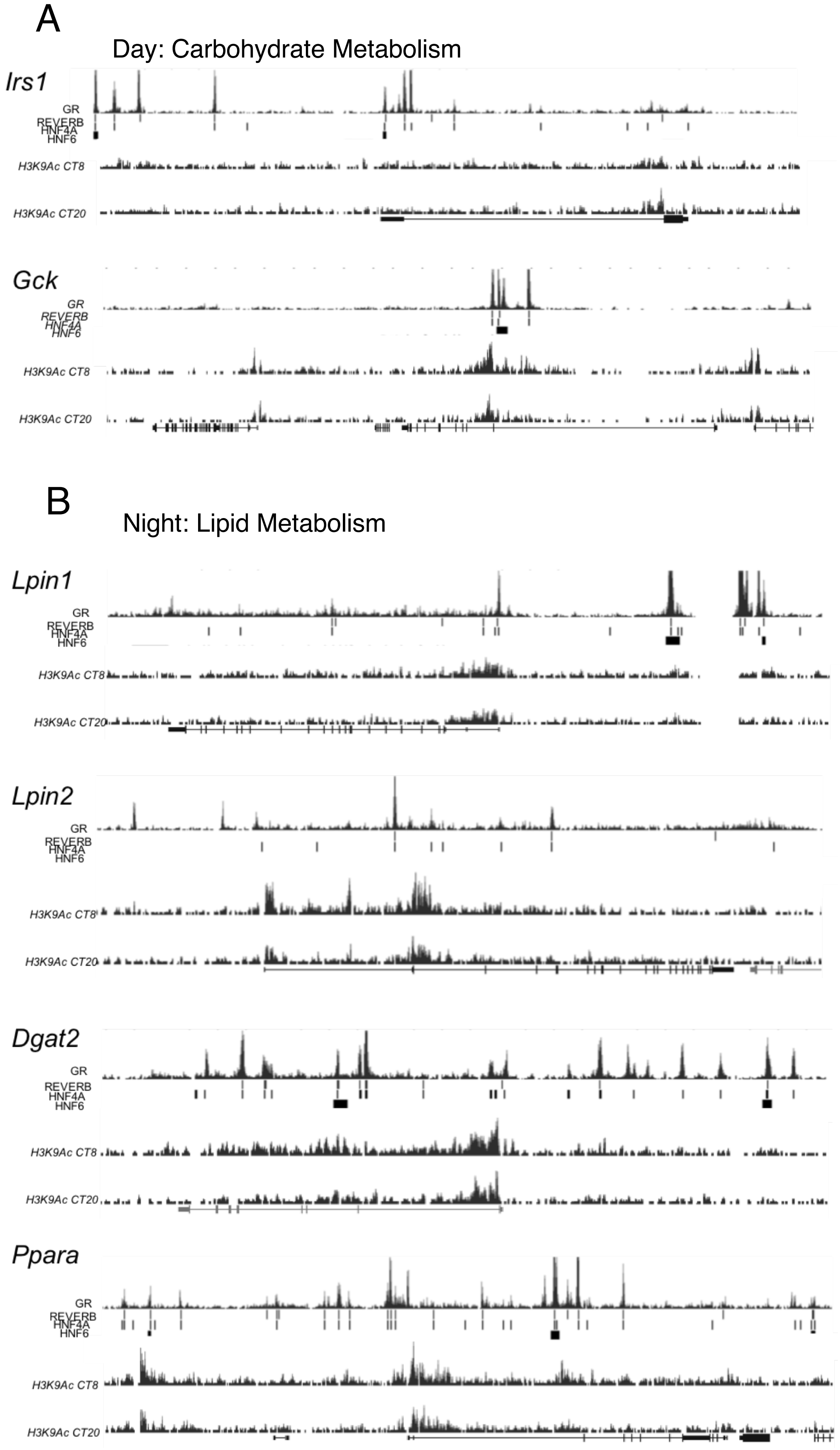

Figure S7. GR-REV-ERBa co-binding sites coincide with HNF4a binding. GR, REV-ERBα, HNF4a and HNF6(day and night) genomic locations are overlaid on GR target genes for carbohydrate metabolism (*Gck* and *Irs1*) (**A**), or target genes for lipid metabolism (*Lpin1, Lpin2, Dgat2* and *Ppara*) (**B**).

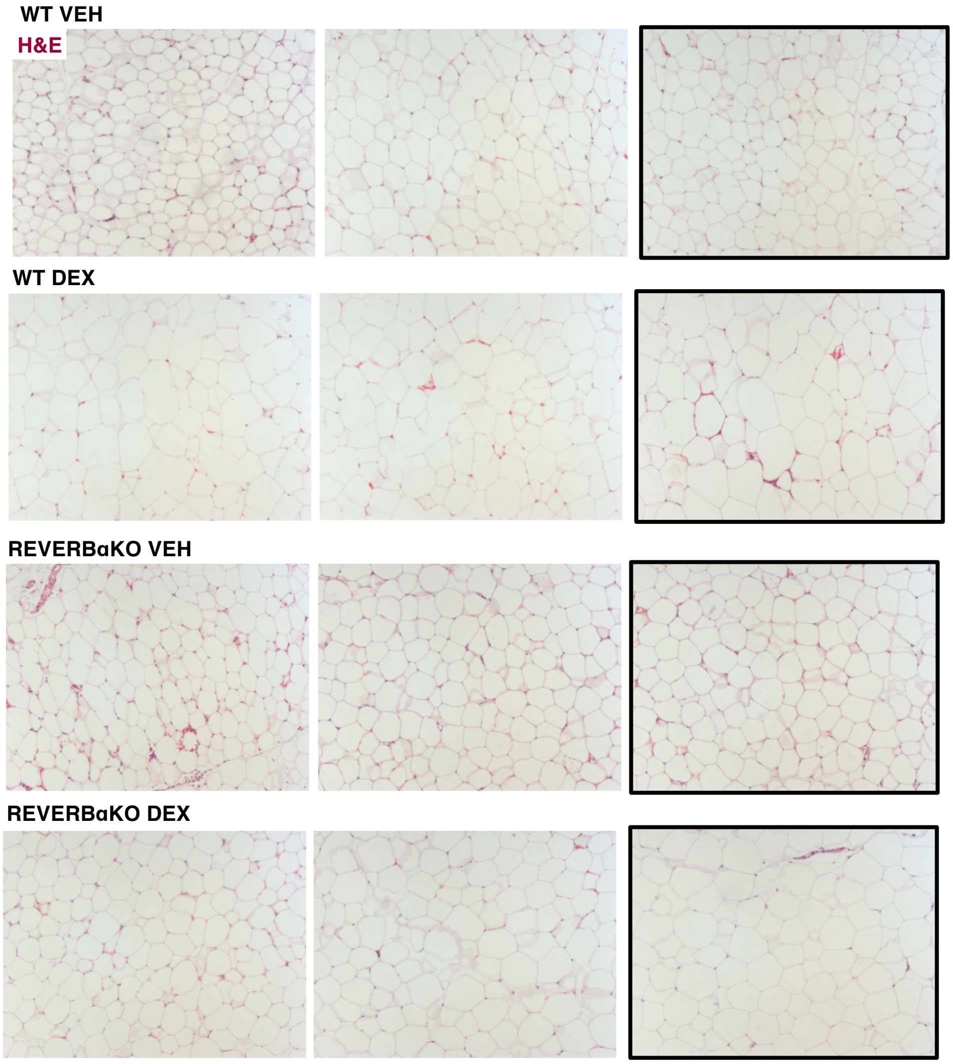

Figure S8. Dex treatment increases adiposity, adipocyte size and heterogeneity in WT but not REV-ERBaKO mice. REV-ERBa knockout and littermate controls were treated with dex or vehicle at ZT6 every 48 hours for 8 weeks. Fat mass was tracked every 14 days and relative change in fat mass for each animal plotted. Means across each group are shown in Fig4C (**A**). Visceral adipose was collected at cull and analysed by H&E. Representative images from three animals per group are shown (**B**). Highlighted images are included in Fig4E.

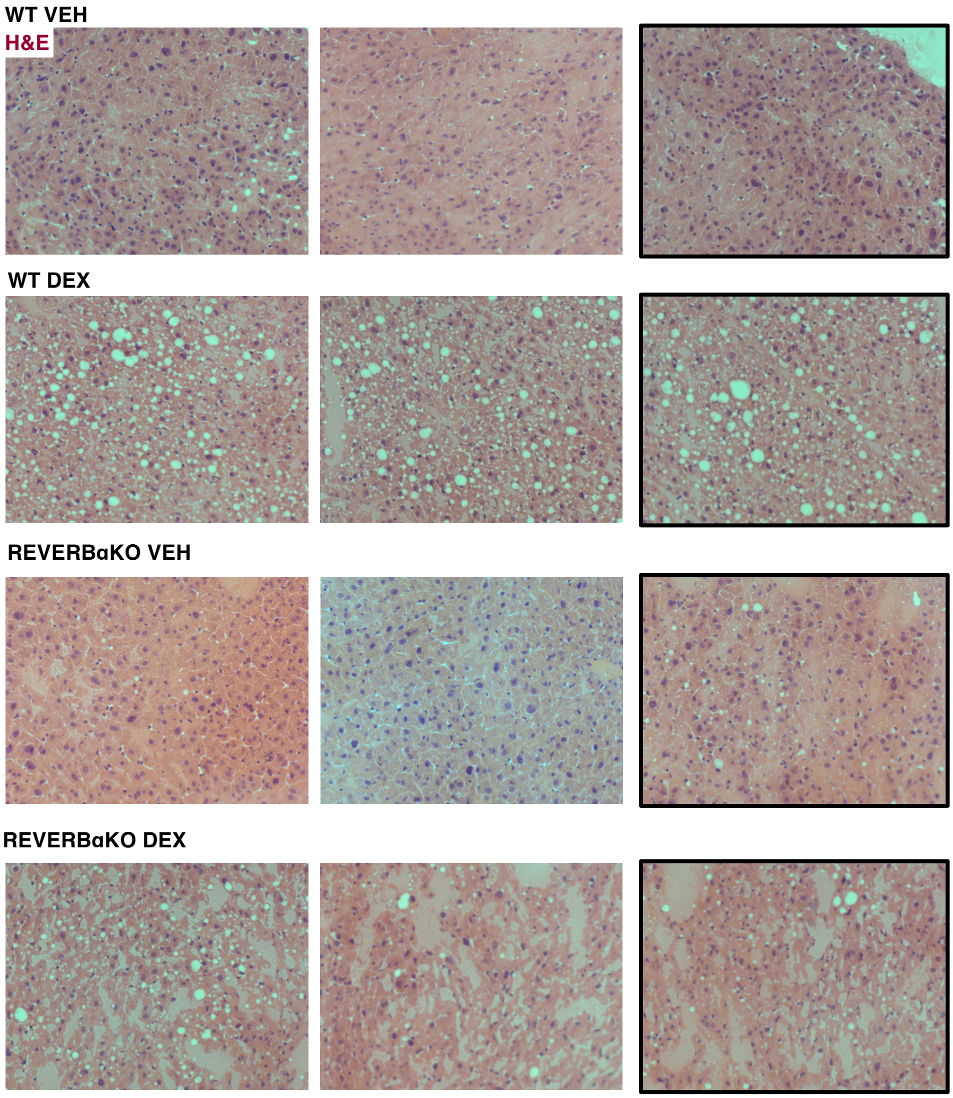

Figure S9. Loss of REV-ERBa protects from GC induced hepatosteatosis. REV-ERBa knockout and littermate controls were treated with dex or vehicle at ZT6 every 48 hours for 8 weeks. Liver was collected at cull and analysed by H&E. Representative images from three animals per group are shown. Highlighted images are included in Fig7E.

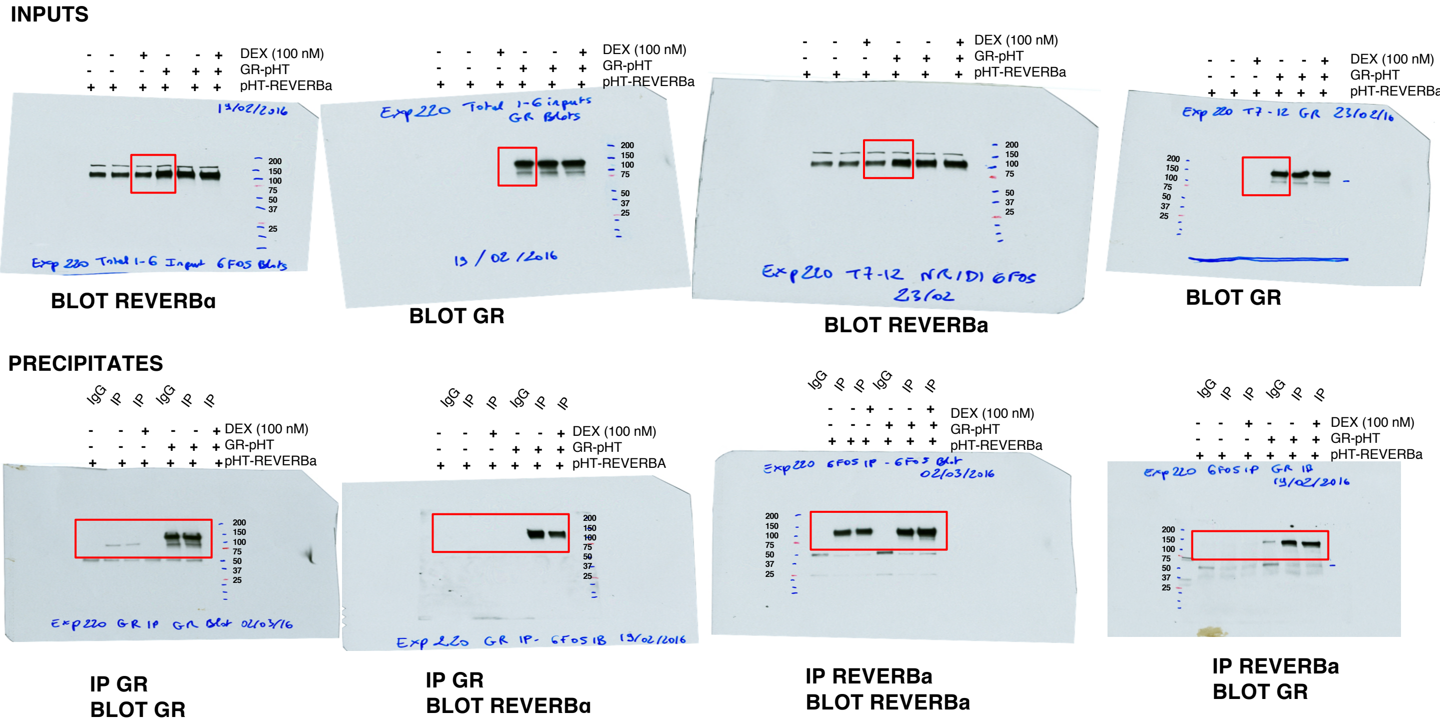

Figure S10. GR interacts with REV-ERBa in vitro. Expanded gel images of GR/REV-ERBa co-immunoprecipitation studies. Full gel scans are shown with cropped images shown in Fig3D highlighted by red boxes. Molecular weight markers are indicated.

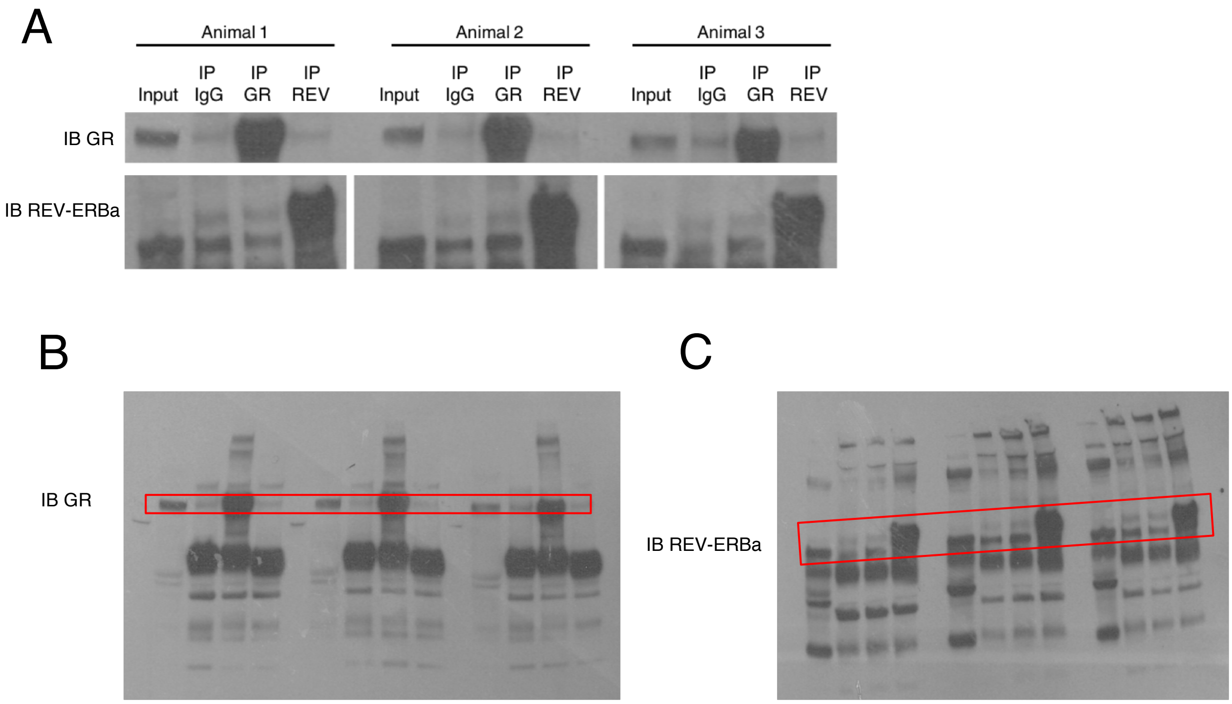

Figure S11. GR and REV-ERBa interaction is not detected by endogenous co-IP *in vivo*. GR IP and REV-ERBa IB, or REV-ERBa IP and GR IB were performed on livers of 3 different animals independently (**A**). Whole blots are shown (**B, C**), red boxes indicate area chosen for A.

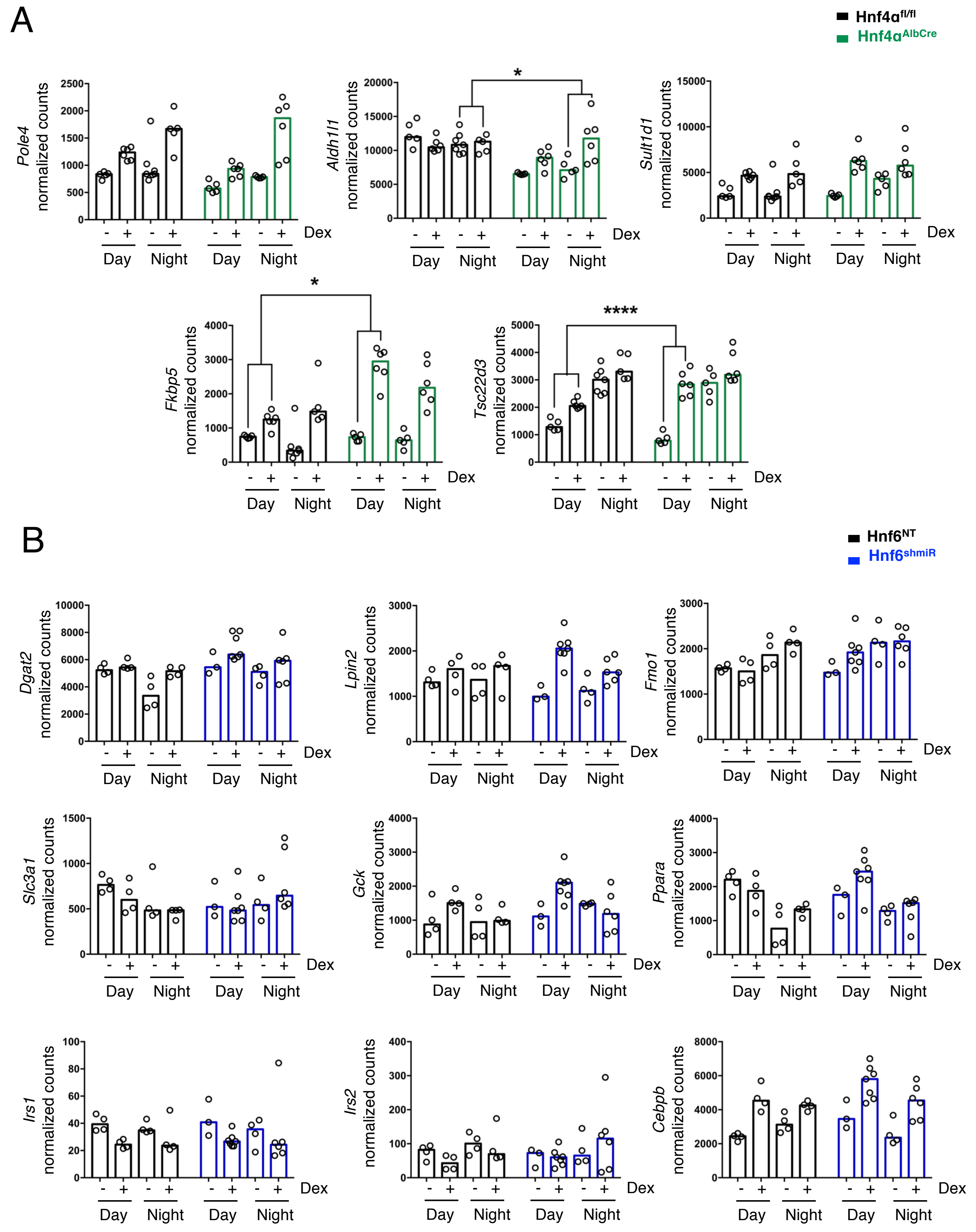

Figure S12. HNF4α and HNF6 control GR time of day gene regulation. $HNF4a^{AlbCre}$ and $HNF4a^{fl/fl}$ controls (N=5-7)(**A**) or C57BL/6J mice were injected with shRNA against *HNF6*, or non-targeting shRNA (N=3-7) (**B**). Mice were treated with 1mg/kg dex at ZT6 or ZT18 and culled 2 hours later. Livers were harvested, and RNA analysed via NanoString. Statistical analysis via LIMMA, $q < 0.05$*, $q < 0.0001$**** data shown as individual mice and median.

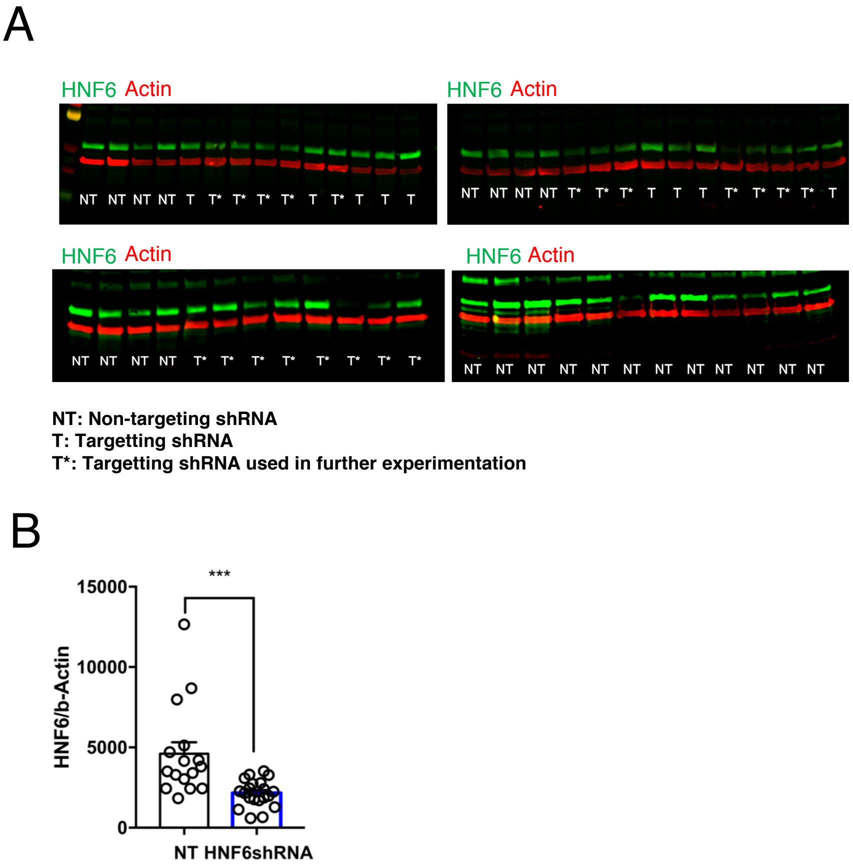

Figure S13. HNF6 knockdown. Whole blots of HNF6 (green) and b-actin (red). NT = non-targeting shRNA, T = targeting. Mice that received targeting shRNA and used in studies are marked with T* (**A**). Knockdown of HNF6 was quantified via comparing HNF6 signal to b-Actin signal (N=16-20) (**B**). Statistical analysis by two-tailed Student's T-test, $p < 0.001$*** data shown as mean ±SEM.

Table S1. KEGG PATHWAY ANALYSIS OF DAY GC TARGETS IN LUNG

PathwayName	#Gene	Statistics
Cytokine-cytokine receptor interaction	25	C=245;O=25;E=2.27;R=11.02;rawP=1.25e-18;adjP=1.54e-16
B cell receptor signaling pathway	14	C=76;O=14;E=0.70;R=19.89;rawP=1.18e-14;adjP=7.26e-13
Hematopoietic cell lineage	13	C=82;O=13;E=0.76;R=17.11;rawP=7.98e-13;adjP=3.27e-11
Metabolic pathways	40	C=1184;O=40;E=10.97;R=3.65;rawP=3.63e-12;adjP=1.12e-10
Natural killer cell mediated cytotoxicity	13	C=125;O=13;E=1.16;R=11.23;rawP=1.91e-10;adjP=4.70e-09
Chemokine signaling pathway	14	C=185;O=14;E=1.71;R=8.17;rawP=2.60e-09;adjP=4.00e-08
Complement and coagulation cascades	10	C=76;O=10;E=0.70;R=14.20;rawP=2.36e-09;adjP=4.00e-08
Pathways in cancer	18	C=325;O=18;E=3.01;R=5.98;rawP=2.20e-09;adjP=4.00e-08
Leishmaniasis	9	C=64;O=9;E=0.59;R=15.18;rawP=8.23e-09;adjP=1.12e-07
Malaria	8	C=46;O=8;E=0.43;R=18.77;rawP=9.86e-09;adjP=1.21e-07
Cell adhesion molecules (CAMs)	11	C=149;O=11;E=1.38;R=7.97;rawP=1.70e-07;adjP=1.75e-06
Osteoclast differentiation	10	C=118;O=10;E=1.09;R=9.15;rawP=1.71e-07;adjP=1.75e-06
MAPK signaling pathway	14	C=268;O=14;E=2.48;R=5.64;rawP=2.72e-07;adjP=2.57e-06
Neuroactive ligand-receptor interaction	14	C=277;O=14;E=2.57;R=5.46;rawP=4.05e-07;adjP=3.32e-06
Staphylococcus aureus infection	7	C=50;O=7;E=0.46;R=15.11;rawP=3.98e-07;adjP=3.32e-06
T cell receptor signaling pathway	9	C=110;O=9;E=1.02;R=8.83;rawP=9.53e-07;adjP=7.21e-06
NOD-like receptor signaling pathway	7	C=57;O=7;E=0.53;R=13.26;rawP=9.97e-07;adjP=7.21e-06
Jak-STAT signaling pathway	10	C=153;O=10;E=1.42;R=7.06;rawP=1.88e-06;adjP=1.28e-05
Toxoplasmosis	9	C=127;O=9;E=1.18;R=7.65;rawP=3.17e-06;adjP=2.05e-05
p53 signaling pathway	7	C=70;O=7;E=0.65;R=10.79;rawP=4.08e-06;adjP=2.51e-05

Table S2. KEGG PATHWAY ANALYSIS OF NIGHT GC TARGETS IN LUNG

PathwayName	#Gene	Statistics
Cytokine-cytokine receptor interaction	27	C=245;O=27;E=2.14;R=12.60;rawP=1.54e-21;adjP=1.60e-19
Hematopoietic cell lineage	16	C=82;O=16;E=0.72;R=22.31;rawP=2.27e-17;adjP=1.18e-15
Primary immunodeficiency	11	C=34;O=11;E=0.30;R=37.00;rawP=4.90e-15;adjP=1.70e-13
Natural killer cell mediated cytotoxicity	16	C=125;O=16;E=1.09;R=14.64;rawP=2.40e-14;adjP=6.24e-13
T cell receptor signaling pathway	15	C=110;O=15;E=0.96;R=15.59;rawP=5.99e-14;adjP=1.25e-12
B cell receptor signaling pathway	12	C=76;O=12;E=0.66;R=18.06;rawP=3.28e-12;adjP=5.69e-11
Chemokine signaling pathway	16	C=185;O=16;E=1.62;R=9.89;rawP=1.10e-11;adjP=1.63e-10
Osteoclast differentiation	13	C=118;O=13;E=1.03;R=12.60;rawP=4.54e-11;adjP=5.25e-10
Graft-versus-host disease	10	C=54;O=10;E=0.47;R=21.18;rawP=4.06e-11;adjP=5.25e-10
Chagas disease (American trypanosomiasis)	12	C=100;O=12;E=0.87;R=13.72;rawP=9.20e-11;adjP=9.57e-10
Leishmaniasis	10	C=64;O=10;E=0.56;R=17.87;rawP=2.38e-10;adjP=2.25e-09
Type I diabetes mellitus	9	C=59;O=9;E=0.52;R=17.44;rawP=2.38e-09;adjP=2.06e-08
MAPK signaling pathway	15	C=268;O=15;E=2.34;R=6.40;rawP=1.96e-08;adjP=1.57e-07
Toll-like receptor signaling pathway	10	C=101;O=10;E=0.88;R=11.32;rawP=2.26e-08;adjP=1.68e-07
Cell adhesion molecules (CAMs)	11	C=149;O=11;E=1.30;R=8.44;rawP=9.58e-08;adjP=6.64e-07
p53 signaling pathway	8	C=70;O=8;E=0.61;R=13.07;rawP=1.90e-07;adjP=1.23e-06
Toxoplasmosis	10	C=127;O=10;E=1.11;R=9.00;rawP=2.01e-07;adjP=1.23e-06
African trypanosomiasis	6	C=32;O=6;E=0.28;R=21.44;rawP=3.24e-07;adjP=1.87e-06
Rheumatoid arthritis	8	C=81;O=8;E=0.71;R=11.29;rawP=5.95e-07;adjP=3.26e-06
Apoptosis	8	C=86;O=8;E=0.75;R=10.64;rawP=9.45e-07;adjP=4.91e-06

Table S3. KEGG PATHWAY ANALYSIS OF DAY GC TARGETS IN LIVER

PathwayName	#Gene	Statistics
Endocytosis	37	C=220;O=37;E=6.17;R=6.00;rawP=2.43e-18;adjP=2.88e-16
Metabolic pathways	92	C=1184;O=92;E=33.21;R=2.77;rawP=3.05e-18;adjP=2.88e-16
Pathways in cancer	40	C=325;O=40;E=9.12;R=4.39;rawP=6.90e-15;adjP=4.35e-13
Ribosome	24	C=119;O=24;E=3.34;R=7.19;rawP=3.23e-14;adjP=1.53e-12
MAPK signaling pathway	35	C=268;O=35;E=7.52;R=4.66;rawP=5.59e-14;adjP=2.11e-12
Focal adhesion	27	C=200;O=27;E=5.61;R=4.81;rawP=1.87e-11;adjP=5.89e-10
Vascular smooth muscle contraction	19	C=123;O=19;E=3.45;R=5.51;rawP=1.78e-09;adjP=4.81e-08
Leishmaniasis	14	C=64;O=14;E=1.80;R=7.80;rawP=2.28e-09;adjP=5.39e-08
Regulation of actin cytoskeleton	25	C=216;O=25;E=6.06;R=4.13;rawP=2.73e-09;adjP=5.73e-08
Axon guidance	19	C=131;O=19;E=3.67;R=5.17;rawP=5.24e-09;adjP=9.90e-08
Chemokine signaling pathway	22	C=185;O=22;E=5.19;R=4.24;rawP=1.46e-08;adjP=2.51e-07
Adipocytokine signaling pathway	13	C=68;O=13;E=1.91;R=6.82;rawP=4.74e-08;adjP=7.47e-07
Wnt signaling pathway	19	C=154;O=19;E=4.32;R=4.40;rawP=7.62e-08;adjP=1.11e-06
Toxoplasmosis	17	C=127;O=17;E=3.56;R=4.77;rawP=1.10e-07;adjP=1.48e-06
Amoebiasis	16	C=116;O=16;E=3.25;R=4.92;rawP=1.70e-07;adjP=2.14e-06
Osteoclast differentiation	16	C=118;O=16;E=3.31;R=4.83;rawP=2.16e-07;adjP=2.55e-06
Insulin signaling pathway	17	C=137;O=17;E=3.84;R=4.42;rawP=3.35e-07;adjP=3.72e-06
Starch and sucrose metabolism	10	C=45;O=10;E=1.26;R=7.92;rawP=3.82e-07;adjP=4.01e-06
NOD-like receptor signaling pathway	11	C=57;O=11;E=1.60;R=6.88;rawP=4.61e-07;adjP=4.59e-06
Bile secretion	12	C=71;O=12;E=1.99;R=6.03;rawP=6.32e-07;adjP=5.69e-06

Table S4. KEGG PATHWAY ANALYSIS OF NIGHT GC TARGETS IN LIVER

PathwayName	#Gene	Statistics
Cysteine and methionine metabolism	3	C=39;O=3;E=0.05;R=56.34;rawP=2.16e-05;adjP=0.0001
Metabolic pathways	8	C=1184;O=8;E=1.62;R=4.95;rawP=0.0002;adjP=0.0005
Protein processing in endoplasmic reticulum	3	C=169;O=3;E=0.23;R=13.00;rawP=0.0016;adjP=0.0027
Steroid hormone biosynthesis	2	C=55;O=2;E=0.08;R=26.63;rawP=0.0026;adjP=0.0032

Table S5 REVERBα DEPENDENT GC TARGETS: LIPID METABOLISM

GENE	DAY	NIGHT
INSIG1	WT ↓	KO ↓
SCARB1	-	KO ↑
CPT1A	-	KO ↑
DGAT2	-	KO ↑
G6PC	-	WT ↓
HNF4A	-	KO ↑
SESN2	-	KO ↑
MIA2	-	KO ↑
ACSL1	KO ↑	KO ↑
CYP2E1	-	KO ↑
LPIN1	WT ↑, KO ↑	KO ↑
LPIN2	KO ↑	KO ↑

Table S6 REVERBα DEPENDENT GC TARGETS: CARBOHYDRATE METABOLISM

GENE	DAY	NIGHT
IRS1	WT ↓	WT ↓
PPP13RG	WT ↓	-
SMEK1	WT ↑	-
PPP1R3B	WT ↑	-
IRS2	WT ↓	-
SORBS1	WT ↓	-
PDK4	WT ↑	-
NR1D1	WT ↓	-
GCK	WT ↑	-
FOXO1	KO ↑	KO ↑
ACER2	WT ↑	-
PGC1A	KO ↑	KO ↑

Table S7 Primer Sequences

GENE	PRIMERS
β-Actin	F-AGGTCATCACTATTGGCAACGA
	R-CACTTCATGATGGAATTGAATGTAGTT
EFNA1	F-GTGGAGAAGCCTGTGGGAAC
	R-GTGTGTATCGCTCCATGGCT
Wt1	F-CACGGCACAGGGTATGAGAG
	R-GTTGGGGCCACTCCAGATAC
Aldh1b1	F-AGCCTCTGTTCAAGTTCAAG
	R-CCTTAAAGCCTCCGAATGG
DIO1	F-GTGGTGGACACAATGCAGAAC
	R-ACGATTGGGTCTATAAGTGGC
GAPDH (Genomic)	F-CAAGAAACAGGGGAGCTGAG
	R-TTGGGTTGTACATCCAAGCA
Mt ND1 (Genomic)	F-GGATCCGAGCATCTTATCCA
	R-GGTGGTACTCCCGCTGTAAA
IL-6	F-GCTACCAAACTGGATATAATCAGGA
	R-CCAGGTAGCTATGGTACTCCAGAA
DUSP1	F-GTGCCTGACAGTGCAGAATC
	R-CACTGCCCAGGTACAGGAAG
TAT (ChIP)	F- CGCAAACAACAGGAAGCCTAA
	R-CATGACACCCAAAAGCCTCTC
Lpin1 (ChIP)	F-TTCTTCCCCTGATGTGCCTG
	R-GCAGGATGTGGGAGTGCTAA

Table S8 Nanostring Codeset – Circadian Control Genes

Gene ID	Gene name	Genotype x Treatment (FDR<0.05)
NM_007771	Cry1	
NM_011066	Per2	
NM_145434	Nr1d1	
NM_011584	Nr1d2	
NM_007715	Clock	
NM_007489	Arntl	

Table S9 Nanostring Codeset – Nuclear Receptor Control Genes

Gene ID	Gene name	Genotype x Treatment (FDR<0.05)
NM_008173	Nr3c1	
NM_009327	Hnf1a	
NM_008261	Hnf4a	HNF4 ZT6
NM_008262	Onecut1	
NM_194268	Onecut2	
NM_001291065	Foxa2	
NM_009883	Cebpb	HNF4 ZT6
NM_009473	Nr1h2	

Table S10 Nanostring Codeset – Gc Responsive Genes

Gene ID	Gene name	Genotype x Treatment (FDR<0.05)
NM_001077364	Tsc22d3	HNF4 ZT6
NM_010220	Fkbp5	HNF4 ZT6
NM_029083	Ddit4	
NM_010107	Efna1	HNF4 ZT18
NM_144783	Wt1	
NM_028270	Aldh1b1	
NM_001190466	Dact1	
NM_008904	Ppargc1a	
NM_133249	Ppargc1b	
NM_028696	Nabp1	
NM_007824	Cyp7a1	
NM_016771	Sult1d1	HNF6 ZT6

Table S11 Nanostring Codeset – Loading Controls

Gene ID	Gene name	Genotype x Treatment (FDR<0.05)
NM_009735	B2m	
NM_007393	Actb	
NM_007907	Eef2	

Table S12 Nanostring Codeset – REVERBa-GR Co-binding Genes

Gene ID	Gene name	Genotype x Treatment (FDR<0.05)
NM_011400	Slc2a1	
NM_172659	Slc2a6	
NM_153526	Insig1	
NM_026384	Dgat2	HNF4 ZT6
NM_001291835	Alas1	
NM_007408	Plin2	
NM_020568	Plin4	
NM_011144	Ppara	HNF4 ZT6
NM_011146	Pparg	
NM_010570	Irs1	
NM_001081212	Irs2	
NM_010292	Gck	HNF4 ZT18
NM_001130412	Lpin1	HNF4 ZT6
NM_001164885	Lpin2	HNF4 ZT6
NM_009205	Slc3a1	HNF4 ZT18
NM_010231	Fmo1	HNF4 ZT6
NM_025882	Pole4	HNF6 ZT6
NM_001356412	Aldh1l1	HNF4 both times, HNF6 ZT6
NM_026534	Ubxn2b	
